# Novel selective strategies targeting the BCL-2 family to enhance clinical efficacy in *ALK*-rearranged non-small cell lung cancer

**DOI:** 10.1038/s41419-025-07513-3

**Published:** 2025-03-20

**Authors:** Fernando Martín, Clara Alcon, Elba Marín, Paula Morales-Sánchez, Albert Manzano-Muñoz, Sherley Díaz, Mireia García, Josep Samitier, Albert Lu, Alberto Villanueva, Noemí Reguart, Cristina Teixido, Joan Montero

**Affiliations:** 1 Institute for Bioengineering of Catalonia (IBEC), Barcelona Institute of Science and Technology (BIST), Barcelona, Spain; 2 Networking Biomedical Research Center in Bioengineering, Biomaterials and Nanomedicine (CIBER-BBN), Madrid, Spain; 3 Department of Biomedical Sciences, Faculty of Medicine and Health Sciences, University of Barcelona, Barcelona, Spain; 4 Division of Medical Oncology, Hospital Clínic, Barcelona, Spain; 5 Translational Genomics and Targeted Therapies in Solid Tumors, August Pi i Sunyer Biomedical Research Institute (IDIBAPS), Barcelona, Spain; 6 Unitat funcional de Tumors Toràcics, Hospital Clínic, Barcelona, Spain; 7 Department of Pathology and CORE Molecular Biology Laboratory, Hospital Clínic, Barcelona, Spain; 8 Department of Electronics and Biomedical Engineering, Faculty of Physics, University of Barcelona, Barcelona, Spain; 9 Chemoresistance and Predictive Factors Group, Program Against Cancer Therapeutic Resistance (ProCURE), Catalan Institute of Oncology (ICO), Oncobell Program, Bellvitge Biomedical Research Institute (IDIBELL), Hospitalet de Llobregat, Spain; 10 Department of Medicine, Faculty of Medicine and Health Sciences, University of Barcelona, Barcelona, Spain

**Keywords:** Targeted therapies, Apoptosis

## Abstract

*ALK* (anaplastic lymphoma kinase) rearrangements represent the third most predominant driver oncogene in non-small cell lung cancer (NSCLC). Although ALK inhibitors are the tyrosine kinase inhibitors (TKIs) with the longest survival rates in lung cancer, the complex systemic clinical evaluation and the apoptotic cell death evasion of drug-tolerant persister (DTP) cancer cells may limit their therapeutic response. We found that dynamic BH3 profiling (DBP) presents an excellent predictive capacity to ALK-TKIs, that would facilitate their use in a clinical setting and complementing the readout of standard diagnostic assays. In addition, we revealed novel acute adaptive mechanisms in response to ALK inhibitors in cell lines and patient-derived tumor cells. Consistently, all our cell models confirmed a rapid downregulation of the sensitizer protein NOXA, leading to dependence on the anti-apoptotic protein MCL-1 after treatment with ALK-TKIs. In some cases, the anti-apoptotic protein BCL-xL may contribute equally to this anti-apoptotic response. Importantly, these acute dependencies could be prevented with BH3 mimetics in vitro and in vivo, blocking tumor adaptation to treatment. Finally, we also demonstrated how dual reactivation of PI3K/AKT and MAPK signaling pathways can impair lorlatinib response, which could be overcome with specific inhibitors of both signaling pathways. In conclusion, our findings propose several therapeutic combinations that should be explored in future clinical trials to enhance ALK inhibitors efficacy and improve the clinical response in a broad NSCLC patient population.

## Introduction

Lung cancer is the leading cause of cancer-related mortality, with a five-year survival rate that barely reaches 22% [1]. Among the patients diagnosed, 85% belong to the histological group known as non-small cell lung cancer (NSCLC) [2]. This high incidence and advances in next-generation sequencing (NGS) led to a broad NSCLC molecular characterization, revealing multiple genomic alterations and potential targets in the development of small-molecule tyrosine kinase inhibitors (TKIs) [3]. Specifically, up to 64% of patients present targetable alterations in driver oncogenes, although not all of them have clinically available treatments [4]. The most frequent are *KRAS* and *EGFR* oncogenes, that are mutated in 30% and 15% of cases, respectively. The third most predominant driver oncogene in NSCLC is *ALK* (anaplastic lymphoma kinase), which is present in around 5% of patients [2, 5]. In contrast to *KRAS* and *EGFR*, constitutive activation of *ALK* usually originates from a fusion with another gene, predominantly *EML4* (90%), which promotes autophosphorylation and downstream activation of MAPK and PI3K/AKT signaling pathways. The first targeted therapy approved for the treatment of *ALK*-positive NSCLC was crizotinib [6]. However, several subsequent next-generation ALK-TKIs, including alectinib, brigatinib, and lorlatinib, demonstrated superior clinical efficacy [7–9], but the discussion over the selection of the optimal first-line therapy is still active [3].

ALK inhibitors are the TKIs conferring longer survival rates in NSCLC, but the response is not durable and treatment options against cell resistance are limited [4]. A hallmark of these drug-tolerant persister (DTP) cancer cells is apoptotic cell death evasion controlled by the BCL-2 family, which plays a key role in the mitochondrial outer membrane permeabilization, considered the point of no return for apoptosis [10, 11]. The BCL-2 family is classified into four groups according to their involvement in this programmed cell death. Briefly, pro-apoptotic effectors BAK and BAX oligomerize and permeabilize the mitochondrial outer membrane. Activators BIM and BID promote BAX and BAK oligomerization. Anti-apoptotic proteins BCL-2, BCL-xL, and MCL-1, among others, prevent apoptosis by sequestering both activator and effector proteins. Finally, sensitizers such as BAD, NOXA, and HRK exert a pro-apoptotic effect by binding to anti-apoptotic members, displacing the activators and effectors sequestered by anti-apoptotic proteins [12, 13]. Therefore, upregulation of anti-apoptotic proteins, together with genetic/epigenetic alterations and post-translational modifications in pro-apoptotic members, predominantly promote cell death resistance and consequent cellular adaptation to anticancer therapy [10, 11]. Nonetheless, these dynamic dependencies could potentially be prevented with BH3 mimetics, novel small molecules that block with high specificity anti-apoptotic proteins [14]. BH3 mimetics’ potential for NSCLC treatment has already been tested in *KRAS* and *EGFR*-mutated tumors [15–17], but alterations in the BCL-2 family in *ALK*-rearranged NSCLC is still not fully understood.

NGS and other molecular analyses made possible to routinely guide clinical decisions, increasing the life expectancy of NSCLC patients by 3–4 years [18–21]. However, these strategies often fail to predict the cytotoxic effect and, especially, dynamic anti-apoptotic resistances to anticancer therapy, since they study the molecular components of dead cancer cells and do not evaluate dynamic responses to perturbations [12, 22]. In contrast, the functional assay dynamic BH3 profiling (DBP) allows the prediction of anticancer therapy cytotoxicity and selective dependencies on anti-apoptotic proteins in DTPs by measuring net changes in mitochondrial apoptotic signaling using synthetic BH3 peptides that mimic the BCL-2 family of proteins [22–28]. We here demonstrate the potential use of DBP to determine the cytotoxicity of ALK inhibitors and the anti-apoptotic programs involved in DTP cancer cell survival in *ALK*-rearranged NSCLC that we believe could impact its treatment in the future.

## Results

### DBP predicts the sensitivity to ALK inhibitors in a representative NSCLC cell lines panel

**Fig. 1:**
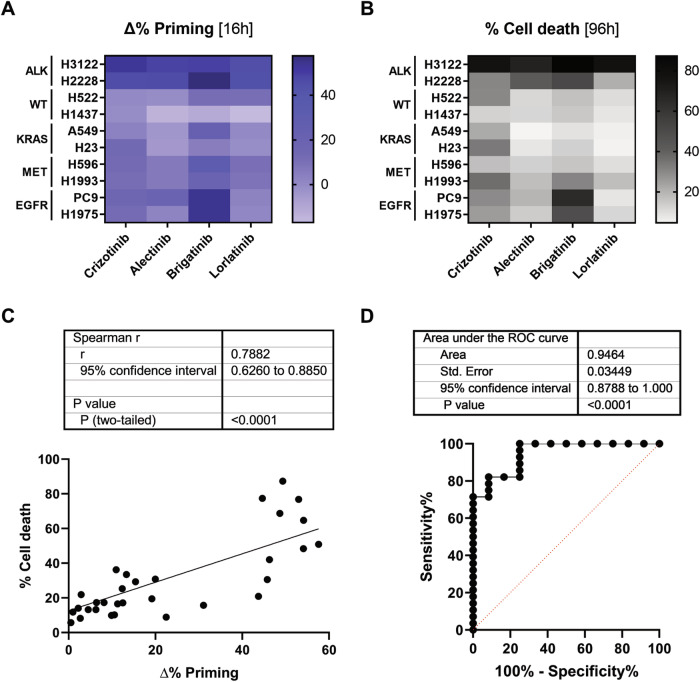
Dynamic BH3 profiling correctly predicts sensitivity to ALK inhibitors in NSCLC cell lines. **A** DBP results in a representative panel of NSCLC (8 cell lines harboring oncogenic driver mutations [*KRAS, EGFR, ALK*, and *MET*] and 2 cell lines without detected alterations in these oncogenes [WT]). All the cell lines were incubated for 16 h with 1 µM of crizotinib, alectinib, brigatinib and lorlatinib. Results expressed as ∆% priming, representing the higher difference in cytochrome c released compared with control condition. Final concentrations of BIM BH3 peptide: 3, 1, 0.3, 0.1, 0.03 and 0.01 µM. **B** Results of cell death assay performed with Annexin V and DAPI staining after 96 h incubation with the same therapies and concentrations. **C** Correlation between % cell death at 96 h and ∆% priming at 16 h. **D** Receiver Operating Characteristic (ROC) curve analysis; treatments that exceeded the threshold of ∆% cell death > 20% were considered responders. The values obtained are from at least three independent experiments.

Current molecular analyses are highly effective in detecting *ALK* rearrangements, but often do not distinguish between different genetic variants of translocation, which may alter the patient’s clinical response [29]. In this sense, we believe that the functional assay DBP could benefit patients by predicting the cytotoxicity of different ALK inhibitors in living tumor cells, complementing the readout of standard diagnostic assays. To demonstrate DBP’s applicability as a biomarker for ALK-TKIs response, we employed a panel of eight NSCLC cell lines that carried the most common oncogenic drivers in NSCLC patients (*KRAS*, *EGFR*, *ALK*, and *MET*), and two other cell lines without detected alterations in these oncogenes [2, 5]. First, we analyzed the increase in Δ% priming using the BIM BH3 peptide after 16 h incubation with crizotinib, alectinib, brigatinib, and lorlatinib. As expected, all ALK-TKIs substantially increased Δ% priming in the two cell lines presenting *ALK* rearrangements, H3122 (*EML4-ALK* gene fusion) [30] and H2228 (*EML4-ALK* and *PTPN3-ALK* gene fusions) [30, 31]. These agents did not increase Δ% priming in the other cell lines, with the notable exception of brigatinib in cell lines presenting *EGFR* mutations (PC9 and H1975), given its inhibitory capacity against this oncogenic protein [32] (Fig. 1A). To validate these DBP predictions, we then confirmed the cytotoxicity of ALK-TKIs in the same panel of cells using Annexin V and DAPI cell death determinations at 96 h (Fig. 1B). Statistical analysis comparing Δ% priming at 16 h and % cell death at 96 h revealed a significant correlation between these two measurements (Fig. 1C). Finally, we carried out a Receiver Operating Characteristic (ROC) Curve analysis to further evaluate the predictive capacity of DBP in NSCLC, reporting an area under the curve (AUC) of 0.94 (Fig. 1D), and demonstrating that DBP was an excellent binary predictor in this representative subset of cell lines and ALK inhibitors.

### The rapid increase in BIM expression determines the response to ALK inhibitors

**Fig. 2:**
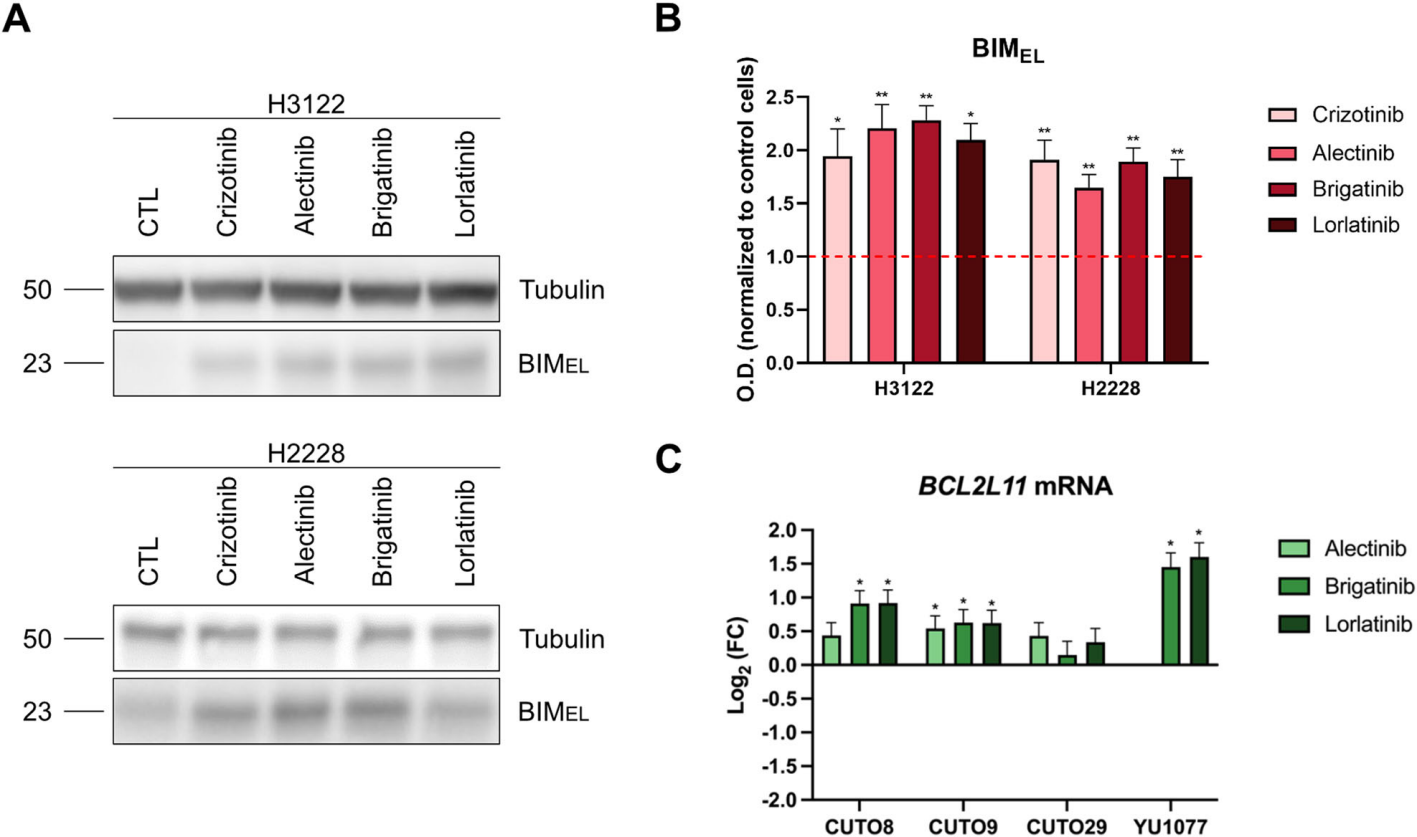
Rapid increase in BIM expression in *ALK*-positive NSCLC cell lines and patient-derived tumor cells in response to ALK inhibitors. **A** Representative images from Western blot analysis of H3122 and H2228 cell lysates after 1 µM of crizotinib, alectinib, brigatinib, and lorlatinib for 16 h. **B** Optical density quantification normalized to tubulin and represented as fold change compared to control. **C** External validation using RNA-seq data of four *EML4-ALK*-positive patient-derived NSCLC cell lines after 24 h of incubation with alectinib, brigatinib, and lorlatinib. Values indicate mean values ± SEM from at least three independent experiments. *p < 0.05 and **p < 0.01. For RNA-seq data, values indicate Log_2_ (FC) ± IfcSE, where IfcSE represents the Standard Error Estimate for the Log_2_ Fold Change Estimate; adjusted *p < 0.05.

The early detection in the increase of Δ% priming is feasible because ALK inhibitors rapidly induced an increase in BIM expression in H3122 and H2228 cell lines (Fig. 2A, B), similarly as previously described for other anticancer agents [33, 34]. Given that accumulation of this activator protein preceded the subsequent apoptotic cell death, treatment-induced BIM accumulation is crucial in cell fate. To investigate if BIM upregulation is also associated with *ALK*-rearranged NSCLC patient-derived cells, we used public RNA-seq data [35] to evaluate changes in *BCL2L11* (BIM gene) mRNA in four *EML4-ALK*-positive patient-derived NSCLC cell lines. All four patient-derived cell lines (CUTO8, CUTO9, CUTO29, and YU1077) exhibited a clear trend in the increase of *BCL2L11* mRNA after 24 h of treatment with alectinib, brigatinib, and lorlatinib (Fig. 2C), correlating with the H3122 and H2228 cell lines data. Finally, to investigate if BIM accumulation also occurs in patients, we performed immunohistochemistry (IHC) analyses of formalin-fixed and paraffin-embedded (FFPE) *ALK*+ NSCLC samples before and during treatment with the ALK inhibitor lorlatinib. Consistent with our in vitro results, tumor tissue prior to ALK inhibition therapy showed negative BIM staining, whereas the histological section removed during lorlatinib treatment showed some areas with a slight increase in BIM expression, although this increment was not as high as expected (Supplementary Fig. S1). Nevertheless, this patient did not survive after 3 months of lorlatinib treatment, which could be explained by the low increase in BIM expression detected. Collectively, these results point to the expression of BIM as a potential predictive biomarker, and support the applicability of DBP in NSCLC patient-derived cells to rapidly identify BIM-mediated apoptotic cell death.

### NOXA downregulation after ALK inhibition leads to anti-apoptotic MCL-1 resistance

The complete clinical response is not always achieved due to apoptotic cell death evasion in DTP cancer cells [10, 11]. Importantly, DBP can identify these dynamic anti-apoptotic protein dependencies using synthetic BH3 peptides that mimic sensitizer BCL-2 family proteins (an increase in priming using the BAD BH3 peptide would indicate BCL-2/BCL-xL tumor dependence; the HRK BH3 peptide, BCL-xL dependence; and the MS1 BH3 peptide, MCL-1 dependence) [12, 22, 27, 36, 37]. These pro-survival adaptations can then be overcome with specific BH3 mimetics such as ABT-199 (or venetoclax, a selective BCL-2 inhibitor) [38], A-1331852 (BCL-xL inhibitor) [39] or S63845 (MCL-1 inhibitor) [40]; or using alternative strategies such as DT2216 (BCL-xL PROTAC) [41] or dinaciclib (a CDK9 inhibitor that downregulates the expression of short half-life RNAs such as *MCL1*) [42–44]. All these agents are now explored in preclinical studies and clinical trials [38–50].

**Fig. 3:**
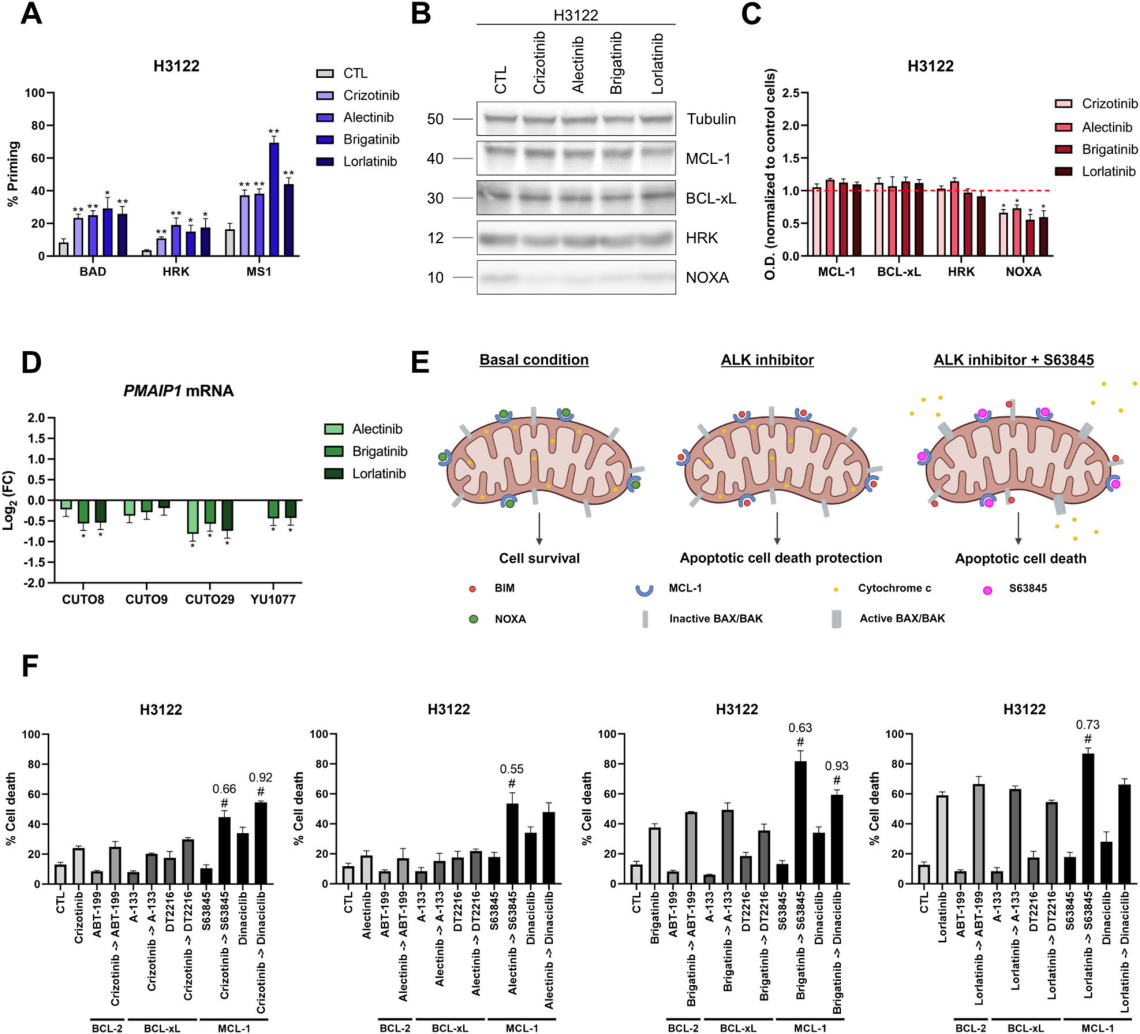
NOXA downregulation induced by ALK inhibition leads to anti-apoptotic MCL-1 resistance. **A** Contribution of each anti-apoptotic protein on the induction of cytochrome c release in response to 1 µM of crizotinib, alectinib, brigatinib, and lorlatinib in H3122 cell line. BAD BH3 peptide correlates with BCL-2/BCL-xL tumor dependence; HRK BH3 peptide, with BCL-xL dependence; and MS1 BH3 peptide, with MCL-1 dependence. Results are expressed as ∆% priming, representing the higher difference in cytochrome c released compared with control condition. The final concentrations of each peptide solution were: 10 µM of BAD BH3 peptide, 100 µM of HRK BH3 peptide, 10 µM or 1 µM of MS1 BH3 peptide. **B** Representative images from Western blot analysis of H3122 cell lysates after 1 µM of crizotinib, alectinib, brigatinib, and lorlatinib for 16 h. **C** Optical density quantification normalized to tubulin and represented as fold change compared to control. **D** External validation using RNA-seq data of four *EML4-ALK*-positive patient-derived NSCLC cell lines after 24 h of incubation with alectinib, brigatinib, and lorlatinib. **E** Graphical scheme of MCL-1 release through NOXA downregulation and sensitization to specific MCL-1 inhibitors. Created in BioRender. Montero, J. (2025) https://BioRender.com/c82p948. **F** Results of cell death assay in H3122 cell line carried out with Annexin V and DAPI staining after 96 h of incubation with crizotinib 0.1 µM, alectinib 0.1 µM, brigatinib 0.01 µM, lorlatinib 0.01 µM, ABT-199 0.1 µM, A-133 0.1 µM, DT2216 0.1 µM, S63845 1 µM, and dinaciclib 0.01 µM. Values indicate mean values ± SEM from at least three independent experiments. *p < 0.05 and **p < 0.01, and # indicates CI < 1 and statistically significant difference (p < 0.05) between the combination treatment and single agents. The value above the # represents the CI. For RNA-seq data, values indicate Log_2_ (FC) ± IfcSE, where IfcSE represents the Standard Error Estimate for the Log_2_ Fold Change Estimate; adjusted *p < 0.05.

We first focused on deciphering the anti-apoptotic adaptations in response to ALK-TKIs in the H3122 cell line using DBP. In this cell line, we observed an increase in % priming with BAD, HRK and MS1 BH3 peptides after treatment with ALK inhibitors for 16 h. However, the highest ∆% priming was clearly obtained with MS1 BH3 peptide (Fig. 3A), suggesting that MCL-1 is the most predominant anti-apoptotic protein involved in the dynamic adaptation against ALK-TKIs in this H3122 cell line, although BCL-xL and/or BCL-2 may play a minor role.

The BCL-2 family represents a complex interactome controlled by dynamic interactions that ultimately mediate the apoptotic response of the cell [14]. To understand the interactions leading to MCL-1 dependence, we analyzed protein expression changes after 16 h of exposure to ALK inhibitors. Surprisingly, MCL-1 expression remained unaltered despite the reported dependence on this anti-apoptotic protein. However, we identified a dramatic decrease in NOXA expression (Fig. 3B, C), a BH3-only sensitizer protein that specifically binds to the anti-apoptotic MCL-1 blocking it. Interestingly, RNA-seq analysis of patient-derived cell lines also revealed a trend in the decrease of *PMAIP1* (NOXA gene) mRNA (Fig. 3D), whereas *MCL1* mRNA levels remained stable (Supplementary Fig. S2). Consistently with other anticancer treatments [27, 51–53], NOXA downregulation after ALK inhibition frees MCL-1 to block BIM accumulation and the apoptotic process engaged by ALK-TKIs, leading to apoptotic cell death protection (Fig. 3E).

Finally, we sequentially combined ALK-TKIs with BH3 mimetics (or aforementioned alternative strategies) to prevent anti-apoptotic adaptations in DTP cancer cells. In this context, we observed a significant reduction in the number of cells when ALK inhibitors were combined with the MCL-1 inhibitor S63845 (Supplementary Fig. S3). These observations were especially notable with crizotinib and brigatinib, that presented limited cytotoxic effect as single agents. In contrast, alectinib and lorlatinib induced a robust reduction in the number of cells, but their combination with S63845 was even more effective. These results demonstrated a reduction in cell proliferation, but did not confirm if these treatments actively killed cancer cells. Since the objective of anticancer therapies is to eliminate malignant cells, we also measured the % cell death by Annexin V and DAPI staining at 96 h. After these combinations, we only found a synergistic and statistically significant Combinatorial Index (CI) in the sequential combination of ALK inhibitors with the MCL-1 inhibitor S63845 or the CDK9 inhibitor dinaciclib (Fig. 3F), correlating with previous results. Therefore, the sequential inhibition of ALK and MCL-1 is able to prevent BIM and MCL-1 binding and restore apoptotic cell death (Fig. 3E).

### Inter-tumor variability in the anti-apoptotic response to ALK inhibitors

**Fig. 4:**
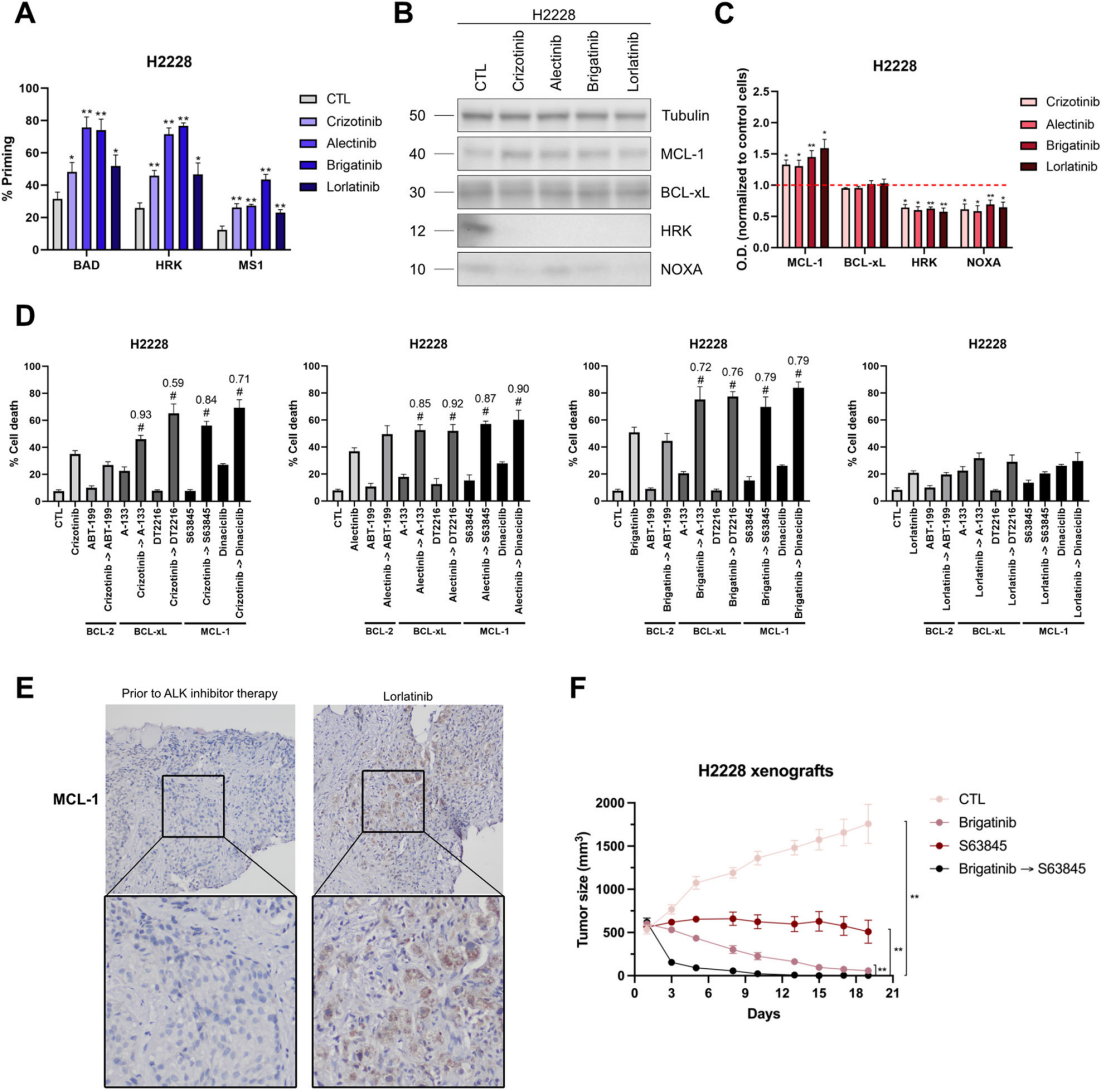
Overcoming anti-apoptotic resistance to ALK inhibitors in vitro and in vivo. **A** Contribution of each anti-apoptotic protein on the induction of cytochrome c release in response to 1 µM of crizotinib, alectinib, brigatinib, and lorlatinib in H2228 cell line. BAD BH3 peptide correlates with BCL-2/BCL-xL tumor dependence; HRK BH3 peptide, with BCL-xL dependence; and MS1 BH3 peptide, with MCL-1 dependence. Results are expressed as ∆% priming, representing the higher difference in cytochrome c released compared with control condition. The final concentrations of each peptide solution were: 10 µM of BAD BH3 peptide, 100 µM of HRK BH3 peptide, 10 µM or 1 µM of MS1 BH3 peptide. **B** Representative images from Western blot analysis of H2228 cell lysates after 1 µM of crizotinib, alectinib, brigatinib, and lorlatinib for 16 h. **C** Optical density quantification normalized to tubulin and represented as fold change compared to control. **D** Results of cell death assay in H2228 cell line carried out with Annexin V and DAPI staining after 96 h of incubation with crizotinib 1 µM, alectinib 1 µM, brigatinib 1 µM, lorlatinib 1 µM, ABT-199 0.1 µM, A-133 0.1 µM, DT2216 0.1 µM, S63845 1 µM, and dinaciclib 0.1 µM. **E** Representative images from immunohistochemistry analysis of formalin-fixed and paraffin-embedded (FFPE) *ALK*+ NSCLC patient samples before and during lorlatinib treatment. 20× magnification. **F** Tumor size (mm^3^) quantification in H2228 xenograft mouse models after treatment with vehicle, brigatinib 10 mg/kg, S63845 20 mg/kg, and brigatinib + S63845. Measurements represent days after initiation of treatment. Values indicate mean values ± SEM from at least three independent experiments. *p < 0.05 and **p < 0.01, and # indicates CI < 1 and statistically significant difference (p < 0.05) between the combination treatment and single agents. The value above the # represents the CI.

To determine whether there is inter-tumor variability in the anti-apoptotic response to ALK-TKIs, we also explored anti-apoptotic adaptations in the H2228 cell line. In contrast with the H3122 cell line, the increase in % priming after ALK inhibitor treatment was quite similar with all three synthetic BH3 peptides (Fig. 4A). Several reports indicate that solid tumors predominantly adapt to therapy through BCL-xL and MCL-1 [54]. In this sense, our analyses with the HRK BH3 peptide (showing a similar ∆% priming as the BAD BH3 peptide) suggested that this increase in ∆% priming indicated a BCL-xL-mediated adaptation. Similar increase was observed with the MS1 peptide, pointing to MCL-1 adaptation and concluding that BCL-xL and MCL-1 are the main anti-apoptotic proteins involved in the resistance to ALK-TKIs in this cell line.

In this case, MCL-1 adaptation was again driven by NOXA downregulation (Fig. 4B, C), emphasizing the significance of this sensitizer protein in the response to ALK inhibitors. In addition, we also detected an upregulation of MCL-1 in the H2228 cell line, further reinforcing this anti-apoptotic adaptation (Fig. 4B, C). As for BCL-xL adaptation, the downregulation of HRK (Fig. 4B, C), a sensitizer BH3-only protein that binds to BCL-xL [12, 51], would liberate this anti-apoptotic protein and sensitize cells to specific BCL-xL inhibitors similarly to the MCL-1:NOXA axis. Although BCL-xL expression did not increase after treatment with ALK-TKIs (Figs. 3B and 4B), the difference in basal levels could also explain the BCL-xL adaptation in the H2228 but not in the H3122 cell line (Supplementary Fig. S2). In patient-derived cell lines, we did not observe a clear trend in *BCL2L1* (BCL-xL gene) nor *HRK* mRNAs (Supplementary Fig. S2), probably due to higher inter-tumor variability compared to the MCL-1:NOXA adaptation.

Lastly, we also evaluated if the sequential combination with BH3 mimetics could prevent these anti-apoptotic adaptations. Most ALK inhibitors (with the exception of lorlatinib) significantly reduced the number of cells. Nevertheless, the combinations with A-1331852 and S63845 were the most effective decreasing the number of cells in all cases (Supplementary Fig. S4), correlating with previous results. To corroborate if these strategies also eliminated cancer cells, we performed cell death analyses. The results showed that most of these dynamic resistances in DTP cancer cells could be overcome by the sequential treatment of ALK inhibitors with A-1331852 (or DT2216) and S63845 (or dinaciclib). However, lorlatinib did not induce cell death even after the inhibition of anti-apoptotic proteins (Fig. 4D), suggesting a potential reactivation of another survival signaling pathway that will be explored in the last section of this study.

### ALK and MCL-1 sequential inhibition enhance cancer treatment in vivo

MCL-1 dependence is the most common anti-apoptotic mechanism observed in our cell lines. If this adaptation to therapy were to occur in patients, MCL-1 inhibitors could improve the treatment of *ALK*-rearranged NSCLC. In this context, we detected an increase in MCL-1 expression in an *ALK*+ NSCLC patient sample after lorlatinib treatment (Fig. 4E). This patient did not respond to therapy, and we hypothesize that MCL-1 could be a potential pro-survival contributor. This finding points to an important role of this anti-apoptotic protein in relapsed tumors, requiring a more extensive analysis to comprehend the implications of this resistance mechanism in cancer patients. In view of these results, we also evaluated whether the combination of ALK inhibitors with S63845 enhanced the treatment in vivo. Similar to cell lines, brigatinib + S63845 caused a significant reduction in the tumor volume compared to single agents in H2228 xenografted tumors (Fig. 4F). Importantly, no treatment significantly reduced the weight of the mouse, demonstrating an acceptable toxicity profile (Supplementary Fig. S5). This anticancer improvement could prevent some relapses with ALK inhibitors, but, since no MCL-1 inhibitors are approved yet for the clinic, further analyses will be required to demonstrate the potential of this combination in patients.

### Reactivation of PI3K/AKT and MAPK signaling pathways impairs lorlatinib cytotoxicity by insufficient BIM accumulation

**Fig. 5:**
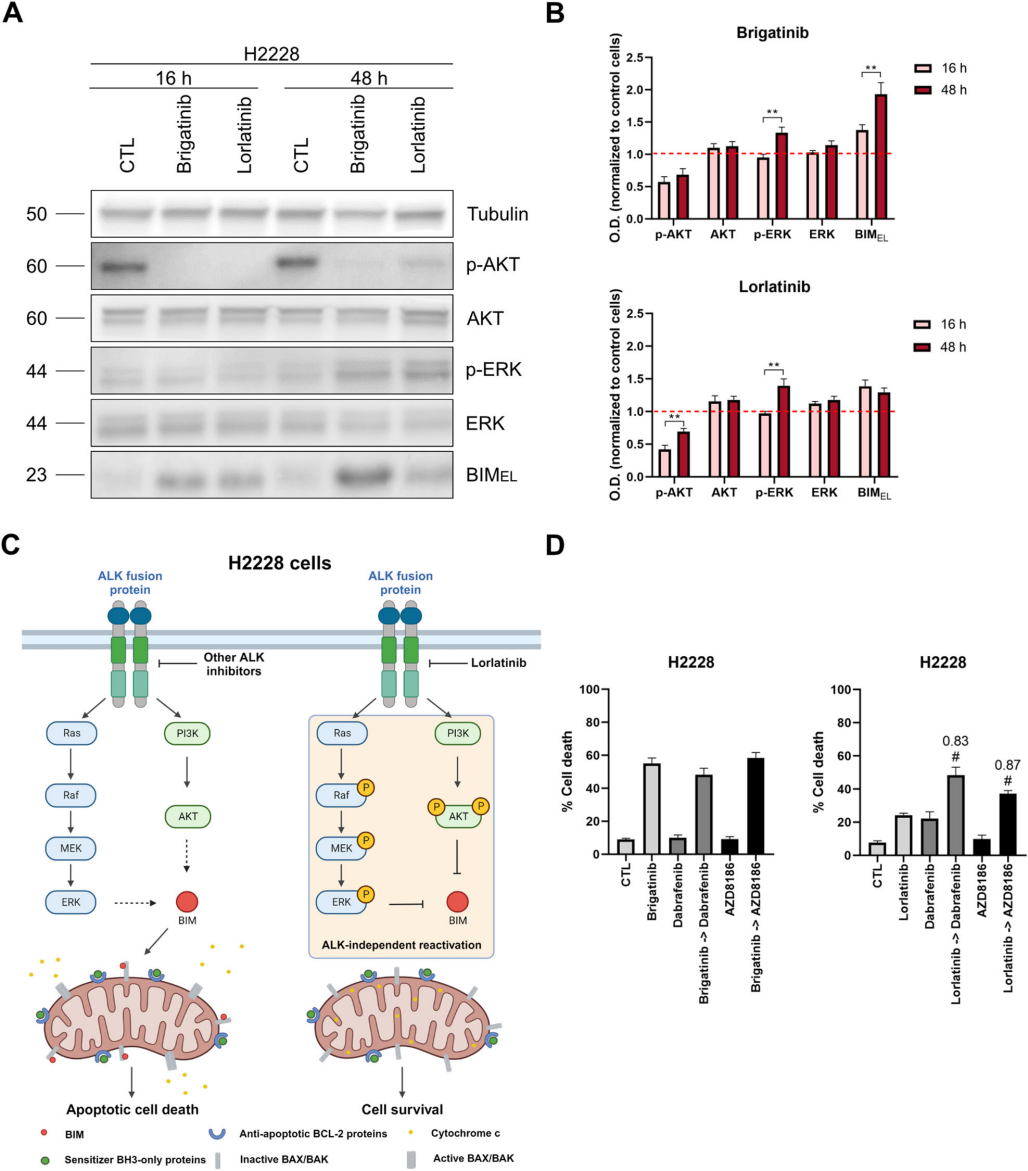
Lorlatinib resistance in the H2228 cell line and use of MAPK and PI3K/AKT pathway inhibitors to overcome it. **A** Representative images from Western blot analysis of H2228 cell lysates after 1 µM of brigatinib and lorlatinib for 16 h and 48 h. **B** Optical density quantification normalized to tubulin and represented as fold change compared to control. **C** Graphical scheme representing the molecular mechanism of lorlatinib resistance in the H2228 cell line. Created in BioRender. Montero, J. (2025) https://BioRender.com/l16s752. **D** Results of cell death assay in H2228 cell line carried out with Annexin V and DAPI staining after 96 h of incubation with brigatinib 1 µM, lorlatinib 1 µM, dabrafenib 1 µM and AZD8186 1 µM. Values indicate mean values ± SEM from at least three independent experiments. *p < 0.05 and **p < 0.01, and # indicates CI < 1 and statistically significant difference (p < 0.05) between the combination treatment and single agents. The value above the # represents the CI.

The mechanism of resistance to lorlatinib observed in previous cell death analyses in the H2228 cell line could mimic some relapses observed in the clinic. Thus, we sought to study alterations in downstream ALK signaling pathways that could provide information on adaptation and survival to lorlatinib. Accordingly, we evaluated changes in protein phosphorylation in downstream PI3K/AKT and MAPK pathways at short (16 h) and longer time points (48 h) of exposure to brigatinib (an effective treatment) and lorlatinib therapies. Immunoblotting analyses revealed a drastic reduction of p-AKT after 16 h of treatment with brigatinib and lorlatinib, which remained constant after 48 h of exposure to brigatinib, but it increased significantly with lorlatinib. Surprisingly, ERK1/2 phosphorylation remained stable through the first 16 h, but incremented significantly after 48 h of treatment with both ALK-TKIs (Fig. 5A, B). These results suggest that the reactivation of both signaling pathways leads to lorlatinib resistance and consequent cell survival, whereas the increase in p-ERK1/2 alone is not sufficient to prevent brigatinib-induced cell death. To further understand the differential cell death between treatments, we also analyzed the expression of BIM, the main activator of apoptosis in this context whose degradation is regulated by both AKT and ERK1/2 signaling [55–58]. Interestingly, upregulation of the MAPK pathway did not prevent BIM accumulation after brigatinib treatment, which significantly increased its expression from 16 h to 48 h. However, lorlatinib treatment initially incremented BIM expression, but did not further increase over time (Fig. 5A, B), consequence of p-AKT and p-ERK1/2 upregulation. This difference in BIM accumulation may explain the observed resistance to lorlatinib, which fails to achieve sufficient BIM accumulation to induce cell death (Fig. 5C).

Understanding lorlatinib resistance is relevant to elucidate potential therapies to prevent relapse in the clinic. To this end, we next inhibited PI3K/AKT and MAPK pathways after 24 h of lorlatinib treatment, before p-AKT and p-ERK1/2 upregulation. Importantly, the sequential combination of lorlatinib with the BRAF inhibitor dabrafenib or the PI3K inhibitor AZD8186 significantly and synergistically increased the % cell death, while the combination with brigatinib did not enhance the cytotoxicity of this ALK inhibitor as single agent (Fig. 5D). Surprisingly, the combination of lorlatinib with dabrafenib or AZD8186 did not restore BIM_EL_ accumulation, but it increased the amount of BIM_L_ and BIM_S_, two isoforms with pro-apoptotic activity (Supplementary Fig. S6). Finally, crizotinib and alectinib induced a similar response to brigatinib, confirming that the dual reactivation of PI3K/AKT and MAPK signaling pathways were specific to lorlatinib (Supplementary Fig. S7). These results confirm that preventing the reactivation of one downstream ALK signaling pathways is sufficient to synergistically enhance the effectiveness of lorlatinib, and we propose its evaluation in future clinical trials.

## Discussion

The therapeutic landscape of NSCLC treatment has drastically changed with the advancement of NGS technologies, which made possible to routinely detect most genomic alterations for personalized treatment decision-making [21]. However, in contrast to other oncogenic proteins, constitutive activation of *ALK* usually originates from a translocation with another gene. Although in most cases it translocates with *EML4*, other fusion partner genes (such as *KIF5B*, *KLC1*, *TGF*, *TPR*, or *PTPN3*) have not yet been fully characterized. This complexity can compromise its systematic evaluation and the therapeutic response to ALK-TKIs [59]. For this reason, fluorescence in situ hybridization (FISH) and IHC have been established as the primary methods for the detection of *ALK* rearrangements, but its accurate application in the clinical setting may be hampered by the need for trained personnel, lack of automation, or the difficulty of identifying some fusion partner genes [29, 59]. In this study, we propose to use the functional assay DBP to complement FISH or IHC techniques by directly evaluating the cytotoxicity of ALK inhibitors on living tumor cells. To this end, we confirmed that DBP was an excellent binary predictor of ALK inhibitor response in NSCLC cell lines comparing DBP predictions with cell death analyses (Fig. 1). Although this study provides a foundation for potential future applications, additional research is required to confirm the predictive capacity of DBP in NSCLC patient samples.

DBP can rapidly predict the cytotoxicity of ALK inhibitors because they induce an increase in the activator protein BIM that subsequently leads to apoptotic cell death. Interestingly, although the molecular information available in *ALK*+ NSCLC patients after ALK inhibitor treatment is limited, we also observed a robust trend in therapy-induced BIM upregulation in patient-derived cancer cells (Fig. 2), in agreement with other studies in *EGFR*-mutant NSCLC that propose BIM as a predictive biomarker of response [60]. These findings, together with its previously demonstrated predictive capacity in murine models and patient biopsies [61], support the use of DBP in a clinical setting to complement current molecular analyses [62].

The precise use of next-generation ALK inhibitors has consolidated them as the TKIs with the longest therapeutic response in NSCLC [4, 63]. However, chronic resistance mechanisms, such as mutations in the kinase domain or off-target alterations, are limiting their therapeutic efficacy [4, 5, 18]. Several studies have shown that chronic resistance may be partially explained by acute adaptations involving anti-apoptotic BCL-2 family proteins; and their pharmacological inhibition could restore sensitivity to apoptosis and prevent disease progression and long-term resistance [61]. In *KRAS*-mutated NSCLC tumors, Nangia et al. described that concurrent blockade of MEK and MCL-1 synergistically increased cytotoxicity in vitro and in vivo [17]; and in *EGFR*-mutated tumors, Hata et al. revealed that EGFR inhibitor-resistant cells responded to dual inhibition of BCL-xL and BCL-2 in patient-derived tumor cells [16]. Unfortunately, these anti-apoptotic adaptations have been only superficially explored in other driver oncogenes such as *ALK* or *MET* [64, 65]. In this study, we identified the anti-apoptotic MCL-1 adaptation in response to ALK inhibitors in cell lines and patient-derived cancer cells. This MCL-1 dependence was driven by a significant downregulation of the sensitizer NOXA, which frees MCL-1 after treatment with ALK-TKIs to block therapy-induced apoptosis, as previously demonstrated in other solid tumors such as melanoma and rhabdomyosarcoma [25, 27]. In some cases, this anti-apoptotic adaptation can also be strengthened by an increase in MCL-1 expression, or coexist with BCL-xL dependence (Figs. 3 and 4). Although BCL-2 is one of the main anti-apoptotic proteins, especially in hematological malignancies, immunoblotting analyses did not reveal the presence of this protein (data not shown). These findings highlight the elevated variability in response to the same anticancer agents, which might be determined by the basal protein expression, post-translational modifications, *EML4-ALK* variants or the fusion partner gene, among other reasons. This high heterogeneity supports the need for functional assays such as DBP to accurately determine the anti-apoptotic proteins involved in this rapidly-acquired resistance.

A potential strategy typically proposed to prevent these acute adaptations are BH3 mimetics. To date, venetoclax (ABT-199) has already been approved for the clinic, while BCL-xL and MCL-1 inhibitors are showing clinical efficacy but may be associated with undesired secondary effects such as thrombocytopenia or cardiac toxicity, respectively [14]. To avoid potential side effects, we suggested the alternative use of DT2216, a PROTAC targeting BCL-xL for degradation in tumor cells but not in platelets [41, 50], and dinaciclib, a CDK9 inhibitor that potently suppresses MCL-1 [42, 44]. Our initial data supports that most acute anti-apoptotic adaptations can be prevented in vitro and in vivo (Figs. 3 and 4), demonstrating their potential for future clinical trials such as the combination of navitoclax with the MEK inhibitor trametinib (NCT02079740) or with the EGFR inhibitor osimertinib (NCT02520778) in advanced NSCLC tumors.

Importantly, we also observed a strong resistance to lorlatinib in the H2228 cell line. This finding may correlate with some relapses observed in NSCLC patients, so understanding this molecular mechanism would help to improve treatment and clinical response. Interestingly, we revealed a dual reactivation of PI3K/AKT and MAPK pathways after lorlatinib treatment. Despite previous findings suggesting that ERK-dependent phosphorylation did not regulate the pro-apoptotic activity of BIM in hematopoietic cells [66], several studies in other cell types have demonstrated how AKT and ERK proteins regulate BIM expression by directly phosphorylating this activator protein and leading to its ubiquitination and proteasomal degradation [55–58]. Consequently, lorlatinib did not induce enough BIM accumulation to trigger cell death in contrast to other ALK inhibitors (Fig. 5, Supplementary Fig. S7). In NSCLC tumors with other oncogenic alterations, this aberrant activation of PI3K/AKT or MAPK pathways has already been described as a mechanism of resistance to chemotherapy, targeted therapies, and radiotherapy [67–70], but to our knowledge this is the first time that this dual reactivation is described as a resistance mechanism to ALK inhibitors. These discoveries have converged in clinical trials exploring different rational therapeutic strategies, such as the combination of the EGFR inhibitor osimertinib with the MEK inhibitor selumetinib (NCT02143466) or the inhibition of the PI3K/AKT pathway in combination with sunitinib, a multitargeted TKI (NCT00555256). We here demonstrate that the PI3K inhibitor AZD8186 and the BRAF inhibitor dabrafenib synergistically and significantly increased the cytotoxicity of lorlatinib in vitro (Fig. 5). Both inhibitors have been evaluated in NSCLC patients, revealing an acceptable tolerability and safety profile [71–73]. These novel combinations have the potential to prevent lorlatinib resistance in the clinic and significantly reduce mortality in a broad population of NSCLC patients with *ALK* rearrangements.

In conclusion, this study demonstrates an excellent DBP predictive capacity for ALK inhibitors cytotoxicity, which is feasible because ALK-TKIs induce a rapid increase of the activator BIM in cell lines and patient-derived tumor cells, fostering the employment of this functional assay in a clinical setting. Additionally, we revealed the predominant anti-apoptotic MCL-1 adaptation in response to ALK inhibitors in *ALK*+ tumors, although some of them may also acquire BCL-xL dependence. Interestingly, most of these dynamic adaptations can be prevented with BH3 mimetics in vitro and in vivo. Finally, we showed that the dual reactivation of PI3K/AKT and MAPK signaling can lead to lorlatinib resistance in specific cases by insufficient BIM accumulation. Importantly, this resistance can be partially prevented with AZD8186 or dabrafenib. All therapeutic combinations explored in this study have great clinical potential to prevent the accumulation of DTP cancer cells and future relapses, and we believe should be further explored in clinical trials.

## Materials and methods

### Cell lines and targeted therapies

H3122, H522, H1437, A549, H23, H596, PC9, and H1975 cell lines were kindly provided by Dr. Noemí Reguart from August Pi i Sunyer Biomedical Research Institute (IDIBAPS). H2228 and H1993 cell lines were purchased at ATCC (ATCC^®^ CRL-5935^TM^ and ATCC^®^ CRL-5909^TM^, ATCC, Manassas, Virginia, USA). All cell lines were cultured in RPMI 1640 medium (31870, Thermo Fisher, Gibco, Paisley, Scotland) supplemented with 10% heat-inactivated fetal bovine serum (10270, Thermo Fisher, Gibco), 1% of L-glutamine (25030, Thermo Fisher, Gibco), and 1% of penicillin and streptomycin (15140, Thermo Fisher, Gibco). These cells were maintained at 37 °C in a humidified atmosphere of 5% CO_2_ and routinely tested for mycoplasma.

Crizotinib, alectinib, brigatinib, lorlatinib, dinaciclib, and dabrafenib were purchased at Selleckchem (Munich, Germany); ABT-199, A-1331852, S63845 and AZD8186 were obtained from MedChemExpress (Monmouth Junction, NJ, USA); and DT2216 was acquired from AbMole BioScience (Houston, Texas, USA). Targeted therapies were added directly in the culture media at doses (ranged from 0.01 to 1 µM) and time points indicated in each single experiment.

### Dynamic BH3 profiling

Dynamic BH3 profiling was performed as described in previous publications [24, 25]. Briefly, 3 × 10^5^ cells per condition (ALK inhibitors or DMSO) were incubated for 16 h at 37 °C. Cells were then collected, stained with the viability marker Zombie Violet (423113, BioLegend, Koblenz, Germany) for 10 min at room temperature (R.T.), washed with PBS and resuspended in MEB buffer (150 mM mannitol, 10 mM hepes-KOH pH 7.5, 150 mM KCl, 1 mM EGTA, 1 mM EDTA, 0.1% BSA, 5 mM succinate). In parallel, solutions of synthetic BH3 peptides were prepared in MEB with 0.002% digitonin (D141, Sigma-Aldrich). The final concentrations of each peptide solution were: 3, 1, 0.3, 0.1, 0.03, and 0.01 µM of BIM BH3 peptide, 10 µM of BAD BH3 peptide, 100 µM of HRK BH3 peptide, 10 µM or 1 µM of MS1 BH3 peptide, 25 µM of alamethicin (BML-A 150-0005, Enzo Life Sciences, Lörrach, Germany) and DMSO in the control condition. Subsequently, each peptide solution was incubated with cell suspensions in a 96-well plate (3795, Corning, Madrid, Spain) for 1 h at R.T. Finally, cells were fixed with formaldehyde, neutralized with N2 buffer (1.7 M tris base, 1.25 M glycine at pH 9.1) and stained with cytochrome c antibody (Alexa Fluor^®^ 647 anti-Cytochrome c - 6H2.B4, 612310, BioLegend). Values expressed represent the mean of at least three independent experiments performed with a high-throughput Flow cytometry SONY instrument (SONY SA3800, Surrey, United Kingdom) or Cytek^®^ Aurora Spectral Flow cytometer (Cytek Bioscience, Freemont, CA, USA). ∆% priming represents the difference in % priming (cytochrome c release) between untreated and treated cells.

### Cell death assay

Quantification of apoptotic cell death was carried out by Annexin V/DAPI staining. Briefly, 5 × 10^4^ cells per condition were incubated for 96 h at 37 °C. Cells were then collected and stained with fluorescent conjugates of Annexin V (Alexa Fluor^®^ 647 Annexin V, 640912, BioLegend) and DAPI (62248, Thermo Fisher). The values expressed represent the mean of at least three independent experiments performed with a flow cytometry Gallios instrument (Beckman Coulter, Nyon, Switzerland). Viable cells are Annexin V negative and DAPI negative, and % cell death was calculated as 100 - % viable cells.

### Proliferation assay

Cell proliferation was evaluated by measuring the number of alive cells using flow cytometry. The values expressed represent the mean of at least three independent experiments performed with a flow cytometry BD LSRFortessa^TM^ Cell Analyzer (BD Biosciences, Franklin Lakes, NJ, USA) or CytoFLEX Flow Cytometer (Beckman Coulter, Brea, CA, USA).

### Protein extraction and quantification

Proteins were extracted with RIPA cell lysis buffer (150 mM NaCl, 5 mM EDTA, 50 mM Tris-HCl pH = 8, 1% Triton X-100, 0.1% SDS, EDTA free Protease Inhibitor Cocktail (4693159001 Roche, Mannkin, Germany)) for 30 min at 4 °C, followed by centrifugation at 16000 × *g* for 10 min. Determination of protein concentration was performed using Pierce^TM^ BCA Protein Assay Kit (23227, Thermo Fisher).

### Immunoblotting

Protein immunodetection was performed as previously described [61]. Briefly, proteins were first separated using SDS-PAGE gels (Mini-Protean TGX Precast Gel 12%, 456–1045, Bio-Rad), and subsequently transferred to PVDF membranes (10600023, Amersham Hybond, Pittsburgh, PA, USA). The membranes were blocked in 5% dry milk dissolved in Tris Buffer Saline with 1% Tween 20 (TBS-T) for 50 min and incubated overnight at 4 °C with the following primary antibodies: rabbit anti-BCL-2 (CST4223, Cell Signaling), rabbit anti-BCL-xL (CST2764, Cell Signaling), rabbit anti-MCL-1 (CST5453, Cell Signaling), rabbit anti-HRK (ab45419, Abcam), rabbit anti-NOXA (CST14766, Cell Signaling), rabbit anti-BIM (CST2933, Cell Signaling), rabbit anti-ERK1/2 (CST137F5, Cell Signaling), rabbit anti-phospho-ERK1/2 (CST4376, Cell Signaling), rabbit anti-AKT (CSTC67E7, Cell Signaling), rabbit anti-phospho-AKT (CST4060S, Cell Signaling), and mouse anti-γ-tubulin (T6557, Sigma-Aldrich). The following day, membranes were incubated with anti-rabbit IgG HRP-linked secondary antibody (CST7074, Cell Signaling) or anti-mouse IgG HRP-linked secondary antibody (CST7076, Cell Signaling) for 1 h. Immunoblots were developed using Clarity ECL Western substrate (1705060, Bio-Rad) and digital imaging was done using the LAS4000 imager (GE Healthcare BioSciences AB, Uppsala, Sweden) or ChemiDoc imager (Bio-Rad Laboratories, Hercules, California, USA). ImageJ software was then used to quantify the integrated optical density of bands, and the bars represent the mean of at least three independent experiments. Full length original western blots are included as Supplementary Information.

### Patient samples

Patient samples were retrospectively included in our study with prior fully informed patient consent and approval from the Local Ethical Committee (HCB/2019/0995 V.4 11/12/2019). The study was conducted in accordance with the principles of the Declaration of Helsinki. Information on therapy and outcomes was collected. Biopsies were obtained from a 51-year-old male patient that had a 5-year history of an adenocarcinoma tumor harboring *ALK* rearrangements. The biopsies were removed before therapy with ALK inhibitors and during treatment with lorlatinib. The patient did not survive more than 3 months from the initiation of therapy with the third-generation ALK inhibitor lorlatinib.

### Immunohistochemistry

Formalin-fixed and paraffin-embedded (FFPE) samples were cut and heated at 60 °C for 40 min. The tissues were treated with histological clearing agent Histoclear II and a decreasing alcohol gradient for deparaffinization and hydration. The slides were then incubated in sodium citrate buffer (10 mM sodium citrate, 0.05% Tween 20, pH 6) for 40 min, washed with TBS 0.025% Triton X-100, and blocked with TBS 1% Normal Serum 1% BSA for 1 h. Tissues were incubated overnight at 4 °C with the following primary antibodies diluted in TBS 1% BSA: rabbit anti-BIM (CST2933, Cell Signaling) and rabbit anti-MCL-1 (CST39224, Cell Signaling). The next day, the slides were washed and incubated with TBS 3% H_2_O_2_ for 20 min. The biotinylated secondary antibody (goat anti-rabbit IgG Antibody (H + L), BA-1000-1.5, Vector Laboratories) diluted in TBS 1% BSA was applied for 1 h. The tissues were then washed and incubated with Vectastain^®^ Elite^®^ ABC-HRP Kit (PK-6100, Vector Laboratories) for 30 min and with DAB substrate (11718096001, Roche, Mannkin, Germany) for 10 min. Nuclei were counterstained with modified Harris hematoxylin. Finally, the sections were dehydrated with an increasing alcohol gradient and the histological clearing agent Histoclear II. BIM and MCL-1 were visualized with brightfield illumination using an Olympus upright microscope (BX43, Olympus) with a 20× objective. Immunohistochemistry (IHC) studies were evaluated by a specialist from the pathology department (Cristina Teixido).

### In vivo experiments

In vivo experiments were conducted on 6- to 8-week-old female Crl:NU-Foxn1nu mice (Envigo). H2228 cell line was injected subcutaneously (3.5 × 10^6^ cells in 150 μl PBS). When tumors reached a homogeneous size around 350–500 mm^3^, mice were randomly allocated into the different treatment groups (n = 7/group): (i) placebo; (ii) S63845 (20 mg/kg diluted in 10% DMSO, 40% PEG300, 5% Tween 80); (iii) brigatinib (10 mg/kg diluted in 50% DMSO, 50% corn oil); and (iv) combined S63845 + brigatinib using the same single doses schedule. Brigatinib was administered by oral gavage (p.o) following a 5-day treatment, 2-day rest schedule for 3 weeks. S63845 was intravenously administrated via tail vein injection (i.v.) for three consecutive days, following a 3-day treatment, 4-day rest schedule for 3 weeks. To minimize the risk of developing drug-induced toxicity in combined treatments, drugs were administered spaced in time (S63845 was administered first, followed by brigatinib 1 h later). Tumors were measured using a caliper every 2–3 days and tumor volume was calculated using the formula v = (w² × l)/2, where l is the longest diameter and w the width. After 21 days of treatment, mice were euthanized and tumors collected, measured, and processed for histologic examination. Animals were housed in a sterile environment, in cages with autoclaved bedding, food, and water. Mice were maintained on a daily 12 h light, 12 h dark cycle. The Institutional Ethics Committees approved the study protocol, and the animal experimental design was approved by the IDIBELL animal facility committee (AAALAC Unit1155) under approved procedure 11973. All experiments were performed in accordance with the guideline for Ethical Conduct in the Care and Use of Animals as stated in The International Guiding Principles for Biomedical Research Involving Animals, developed by the Council for International Organizations of Medical Sciences.

### Statistical analysis

All statistical analyses and graphs were generated with GraphPad Prism9. For ROC curve analysis, treatments that exceeded the threshold of ∆% cell death >20% were considered responders. Drug synergies were established based on the previously described Bliss Independent model [74]. Briefly, the Combinatorial Index (CI) was calculated as CI = ((D_A_ + D_B_)−(D_A_ × D_B_))/D_AB_ (D represents cell death of compound A or B or the combination of both). A synergistic combination was considered when CI < 1 and there was statistical significance (p < 0.05) between the combination regimen and single agents. When both conditions were given, it was represented with # in the Figures. Statistical significance of the results was performed using Student’s t-tail test, considering significant *p < 0.05 and **p < 0.01. SEM represents the Standard Error of the Mean. Finally, external validation of our in vitro results was performed with RStudio using RNA-seq data deposited in ArrayExpress with the accession number E-MTAB-11342, considering significant when adjusted p-value < 0.05. IfcSE represents the Standard Error Estimate for the Log_2_ Fold Change Estimate.

## Supplementary information

Supplementary Data

Supplemental Material

## Data Availability

All data are available from the corresponding author upon reasonable request.
