# Supplementary material for: Novel selective strategies targeting the BCL-2 family to enhance clinical efficacy in ALK-rearranged non-small cell lung cancer
Source: Cell Death Dis. 2025 Mar 20;16(1):194. doi: 10.1038/s41419-025-07513-3 (PMC11926089; doi:10.1038/s41419-025-07513-3)
Supplement: Supplementary file 1 — Supplementary Data [file 41419_2025_7513_MOESM1_ESM.docx]

**SUPPLEMENTARY FIGURES**

**
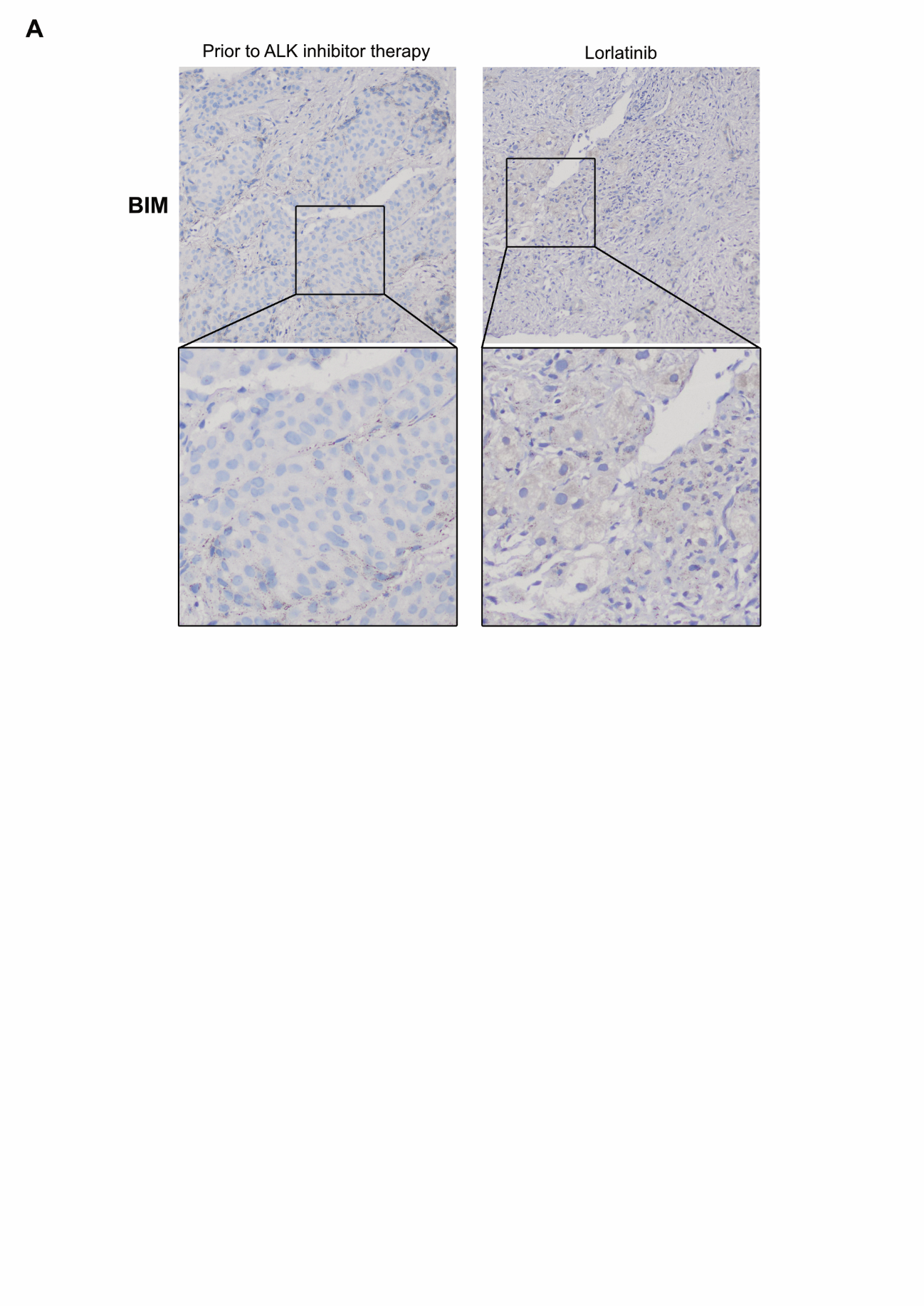
**

***Supplementary Figure S1: Slight increase in BIM expression in a patient sample excised during lorlatinib treatment.*** *(A) Representative images from immunohistochemistry analysis of formalin-fixed and paraffin-embedded (FFPE) ALK+ NSCLC patient samples before and during lorlatinib treatment. 20x magnification.*

***
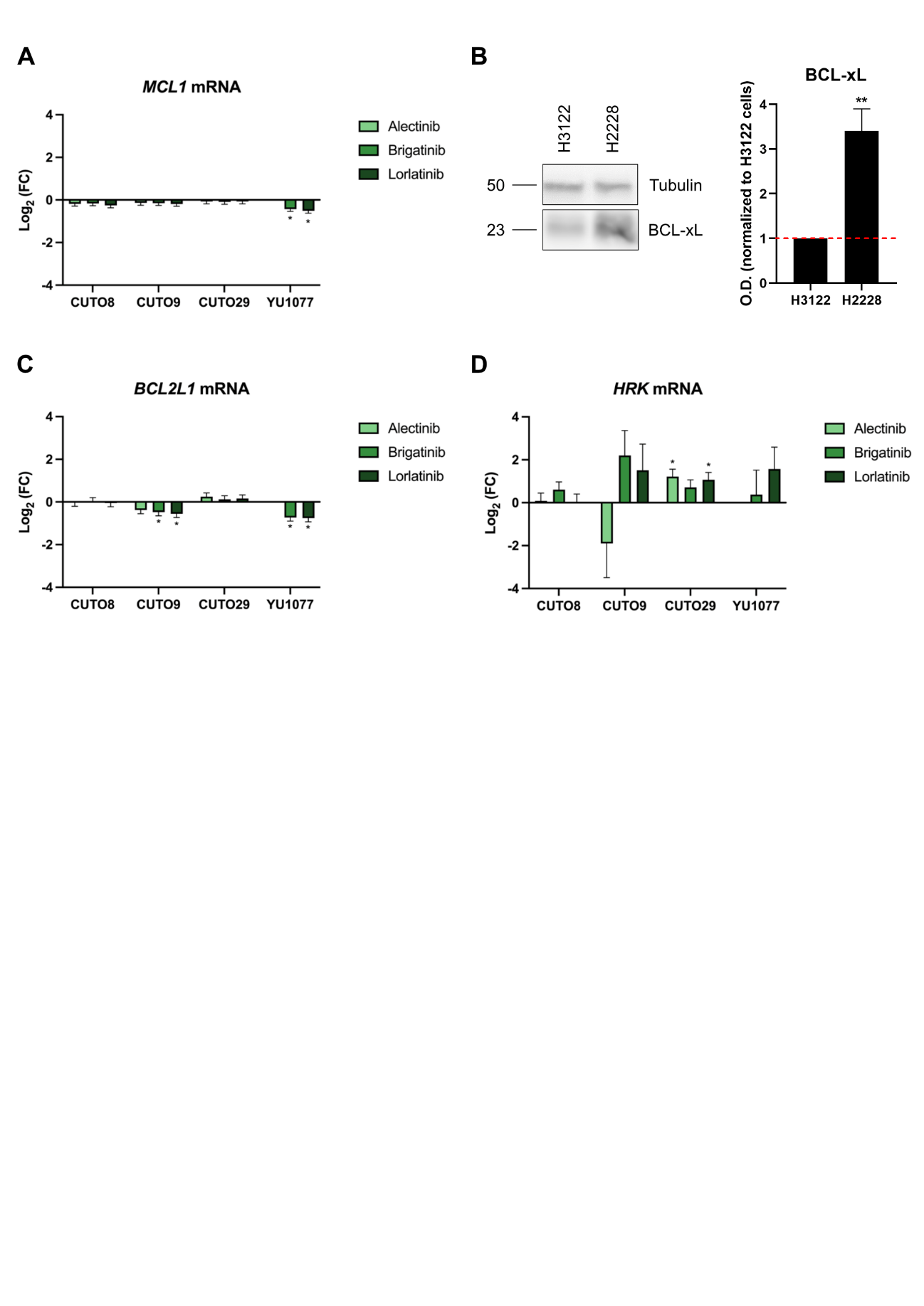
***

***Supplementary Figure S2: Protein or mRNA expression changes of BCL-2 family members after treatment with ALK inhibitors or between NSCLC cell lines.*** *(A, C, D) External validation using RNA-seq data of four EML4-ALK-positive patient-derived NSCLC cell lines after 24 h of incubation with alectinib, brigatinib and lorlatinib. (B) Representative images from Western blot analysis of H3122 and H2228 cell lysates after DMSO for 16 h (left) and optical density quantification normalized to tubulin and represented as fold change compared to control (right). Values indicate mean values ± SEM from at least three independent experiments. **p < 0.01. For RNA-seq data, values indicate Log_2_ (FC) ± IfcSE, where IfcSE represents the Standard Error Estimate for the Log_2_ Fold Change Estimate; adjusted *p < 0.05.*

***
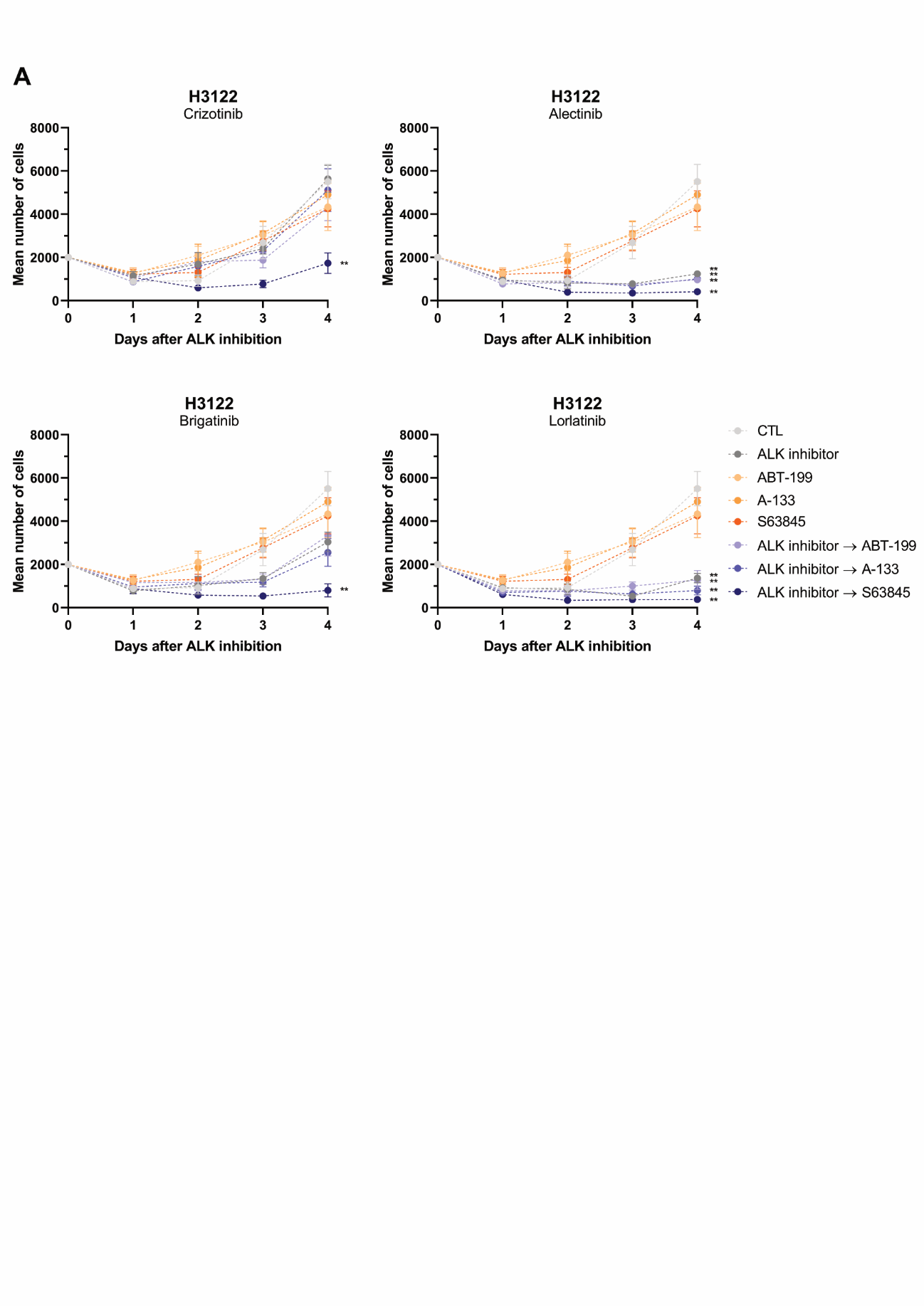
***

***Supplementary Figure S3: Sequential inhibition of ALK and MCL-1 reduced the mean number of cells in the H3122 cell line.*** *(A) Results of proliferation assay in the H3122 cell line after the incubation with crizotinib 0.1 µM, alectinib 0.1 µM, brigatinib 0.01 µM, lorlatinib 0.01 µM, ABT-199 0.1 µM, A-1331852 0.1 µM and S63845 1 µM. Values indicate mean values ± SEM from at least three independent experiments. **p < 0.01 between treatments and control condition.*

***
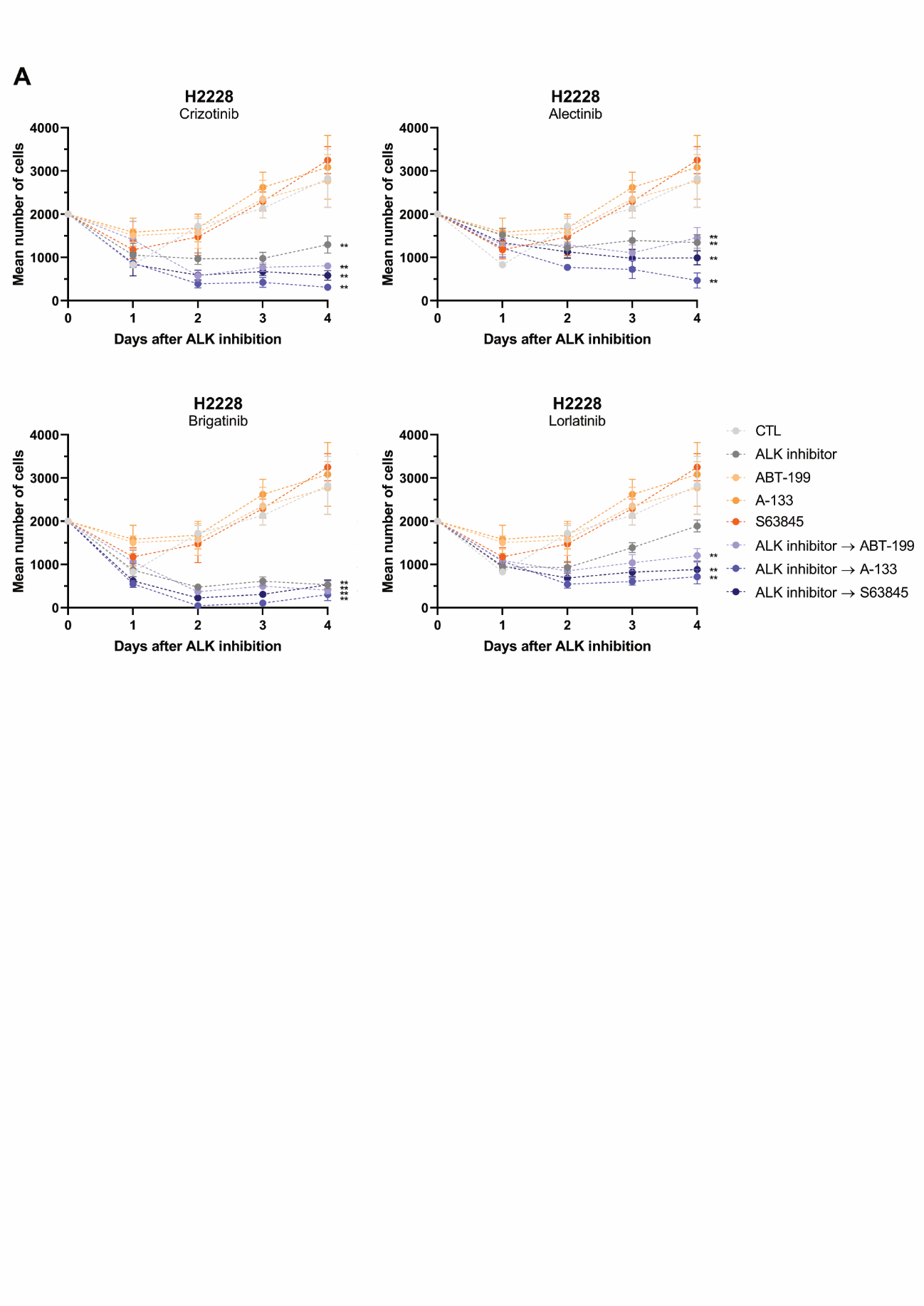
***

***Supplementary Figure S4: Sequential inhibition of ALK and MCL-1 or BCL-xL reduced the mean number of cells in the H2228 cell line.*** *(A) Results of proliferation assay in the H2228 cell line after the incubation with crizotinib 1 µM, alectinib 1 µM, brigatinib 1 µM, lorlatinib 1 µM, ABT-199 0.1 µM, A-1331852 0.1 µM and S63845 1 µM. Values indicate mean values ± SEM from at least three independent experiments. **p < 0.01 between treatments and control condition.*

*
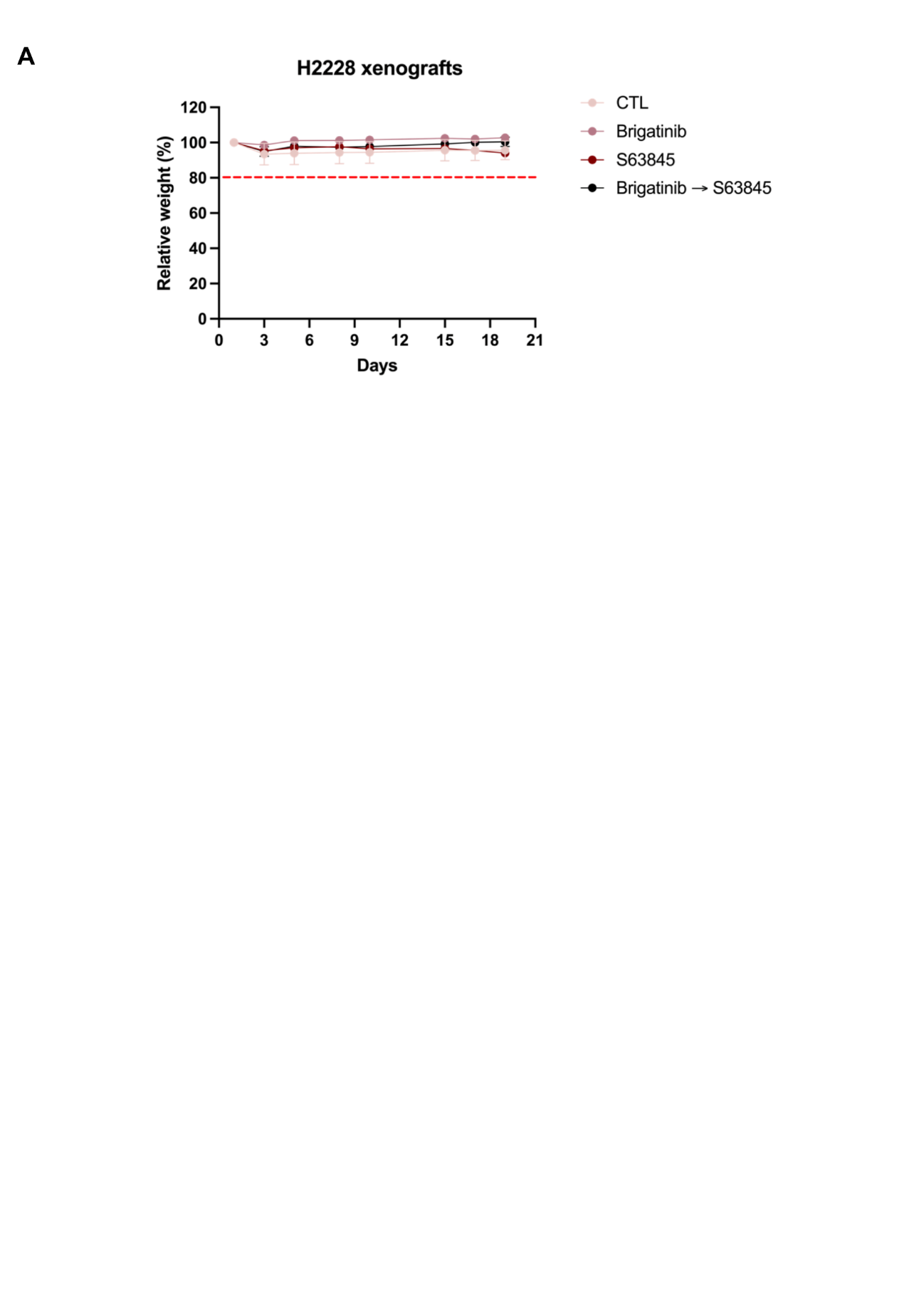
*

***Supplementary Figure S5: No treatment significantly reduced the weight of the mouse, demonstrating an acceptable toxicity profile.*** *(A) Progression of body weight in H2228 xenograft mouse models after treatment with vehicle, brigatinib 10 mg/kg, S63845 20 mg/kg, and brigatinib + S63845. Measurements represent days after initiation of treatment. The red line indicates a 20% reduction from the initial weight, which is associated with therapy toxicity.*

***
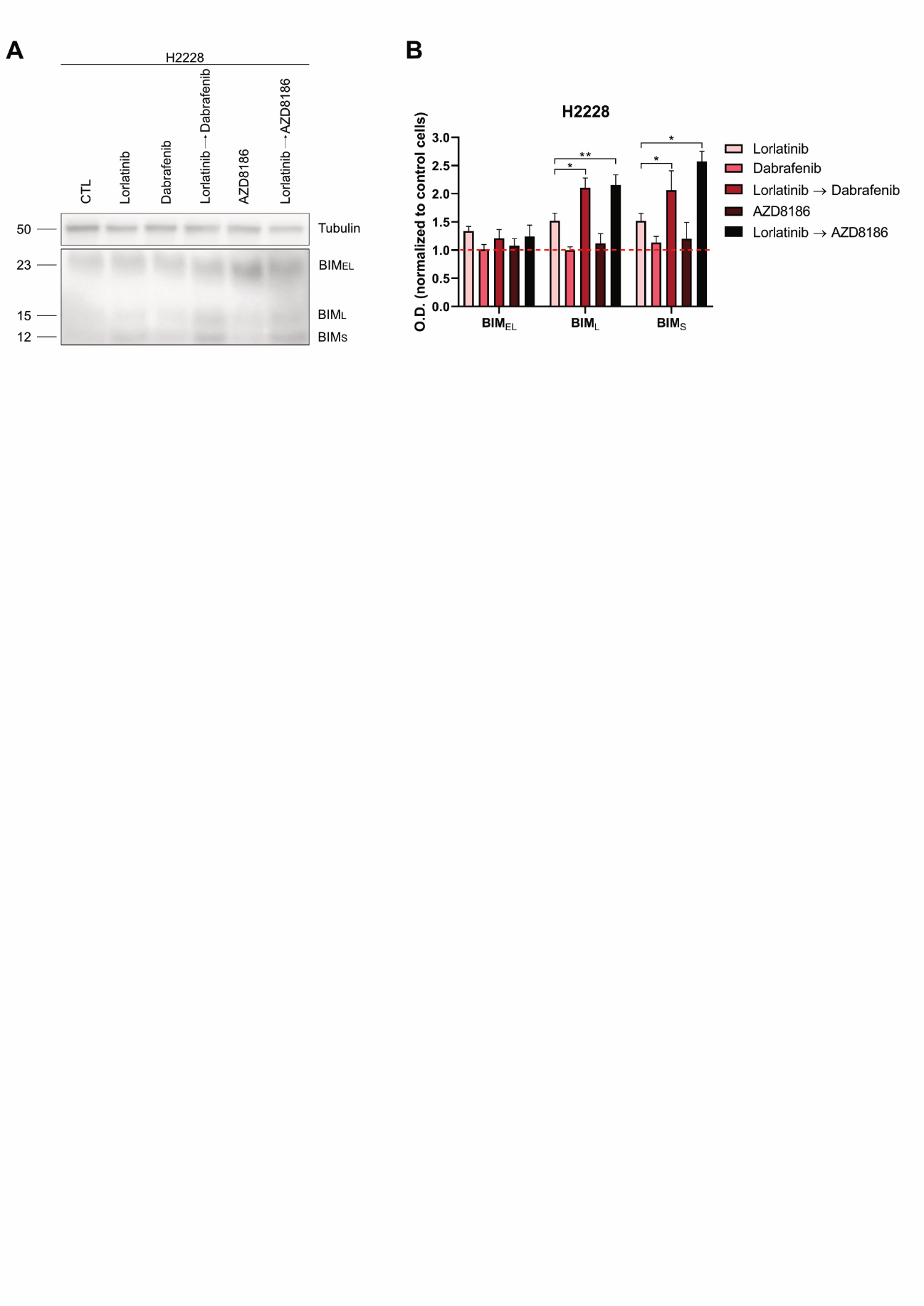
***

***Supplementary Figure S6: Sequential inhibition of ALK and BRAF or PI3K restored the expression of BIM_L_ and BIM_S_.*** *(A) Representative images from Western blot analysis of H2228 cell lysates after 1 µM of lorlatinib, dabrafenib and AZD8186 (and combinations) for 48 h. (B) Optical density quantification normalized to tubulin and represented as fold change compared to control. Values indicate mean values ± SEM from at least three independent experiments. *p < 0.05 and **p < 0.01.*

*
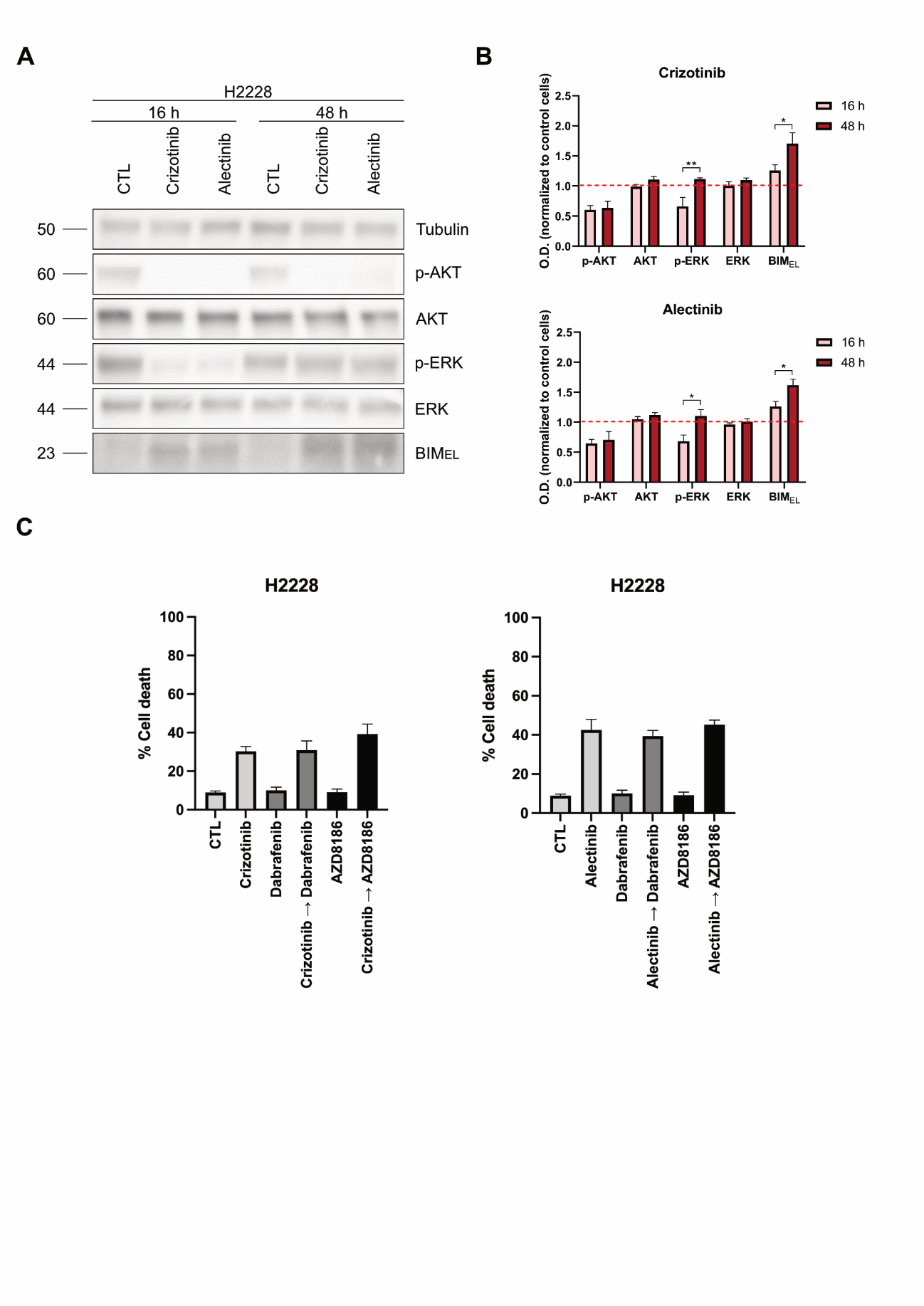
*

***Supplementary Figure S7: Crizotinib and alectinib did not induce dual reactivation of downstream MAPK and PI3K/AKT signaling pathways.*** *(A) Representative images from Western blot analysis of H2228 cell lysates after 1 µM of crizotinib and alectinib for 16 h and 48 h. (B) Optical density quantification normalized to tubulin and represented as fold change compared to control. (C) Results of cell death assay in H2228 cell line carried out with Annexin V and DAPI staining after 96 h of incubation with crizotinib 1 µM, alectinib 1 µM, dabrafenib 1 µM and AZD8186 1 µM. Values indicate mean values ± SEM from at least three independent experiments. *p < 0.05 and **p < 0.01.*
