# Supplementary material for: Novel selective strategies targeting the BCL-2 family to enhance clinical efficacy in ALK-rearranged non-small cell lung cancer
Source: Cell Death Dis. 2025 Mar 20;16(1):194. doi: 10.1038/s41419-025-07513-3 (PMC11926089; doi:10.1038/s41419-025-07513-3)

**ORIGINAL WESTERN BLOTS**

We here provide the full length uncropped original western blots used in the manuscript. As each western blot contains different proteins and conditions, the sections included in the main Figure are marked with a red rectangle.

**Figure 2**

H3122 cell line

Tubulin


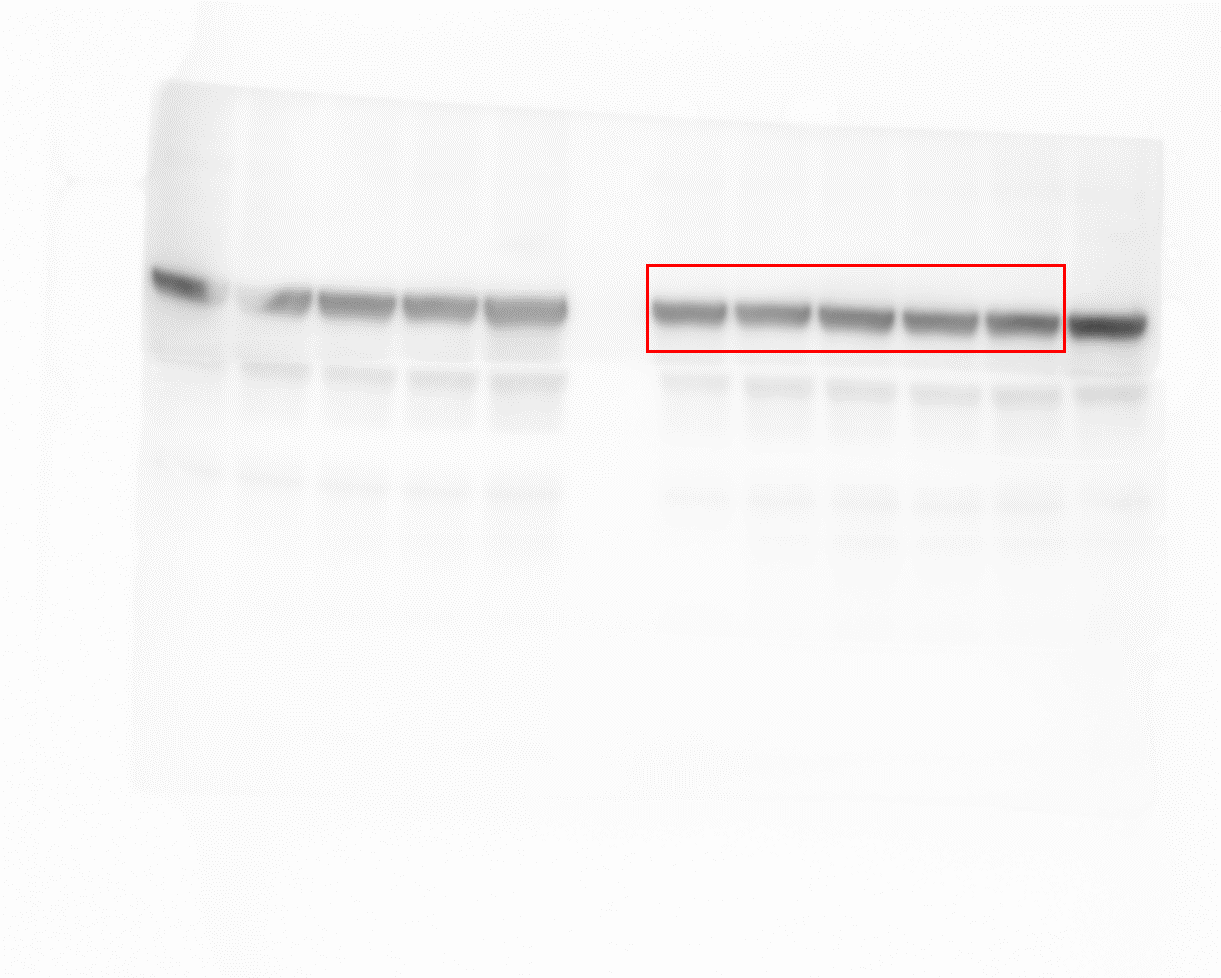


BIM_EL_

_
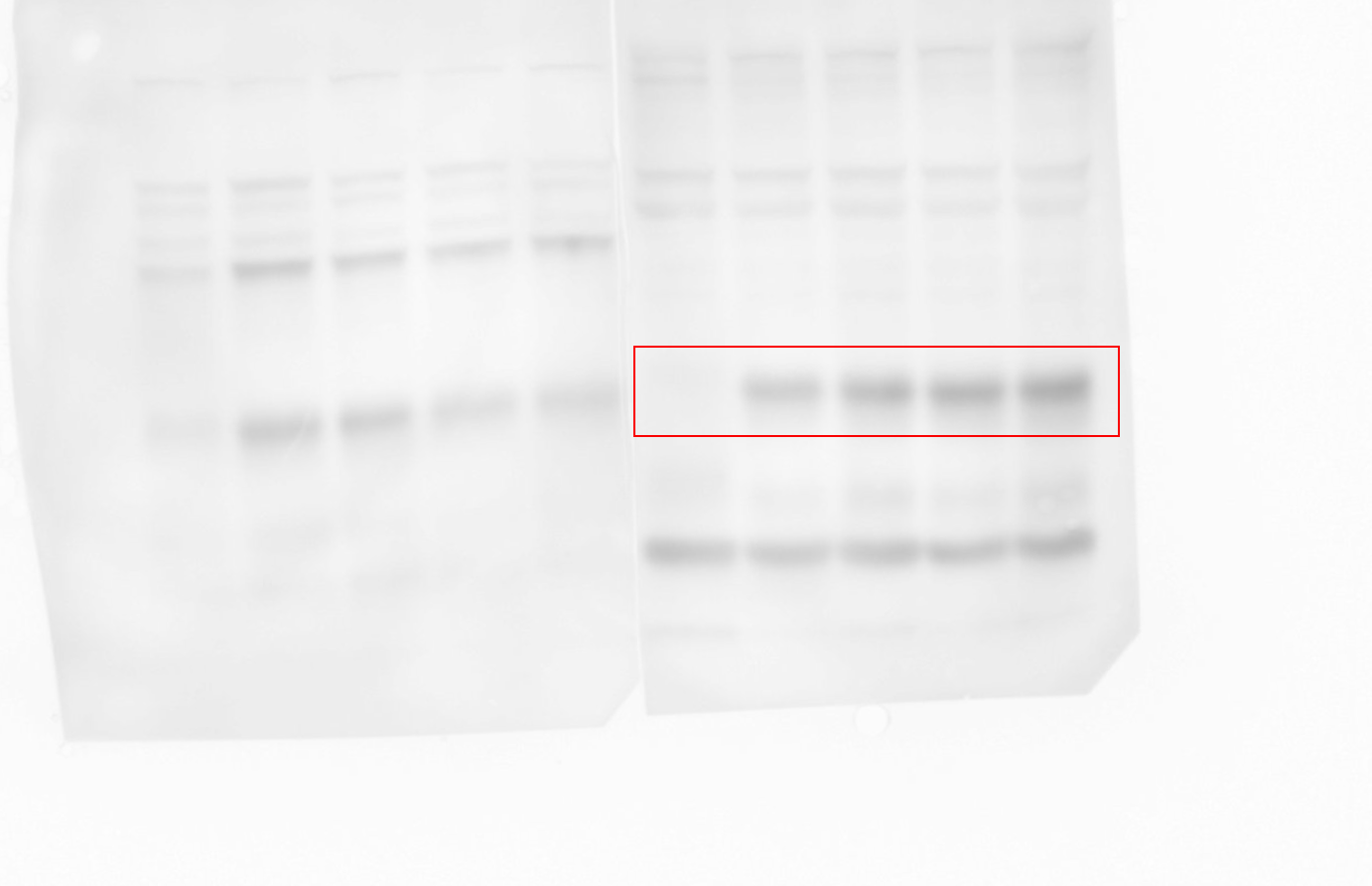
_

H2228 cell line

Tubulin


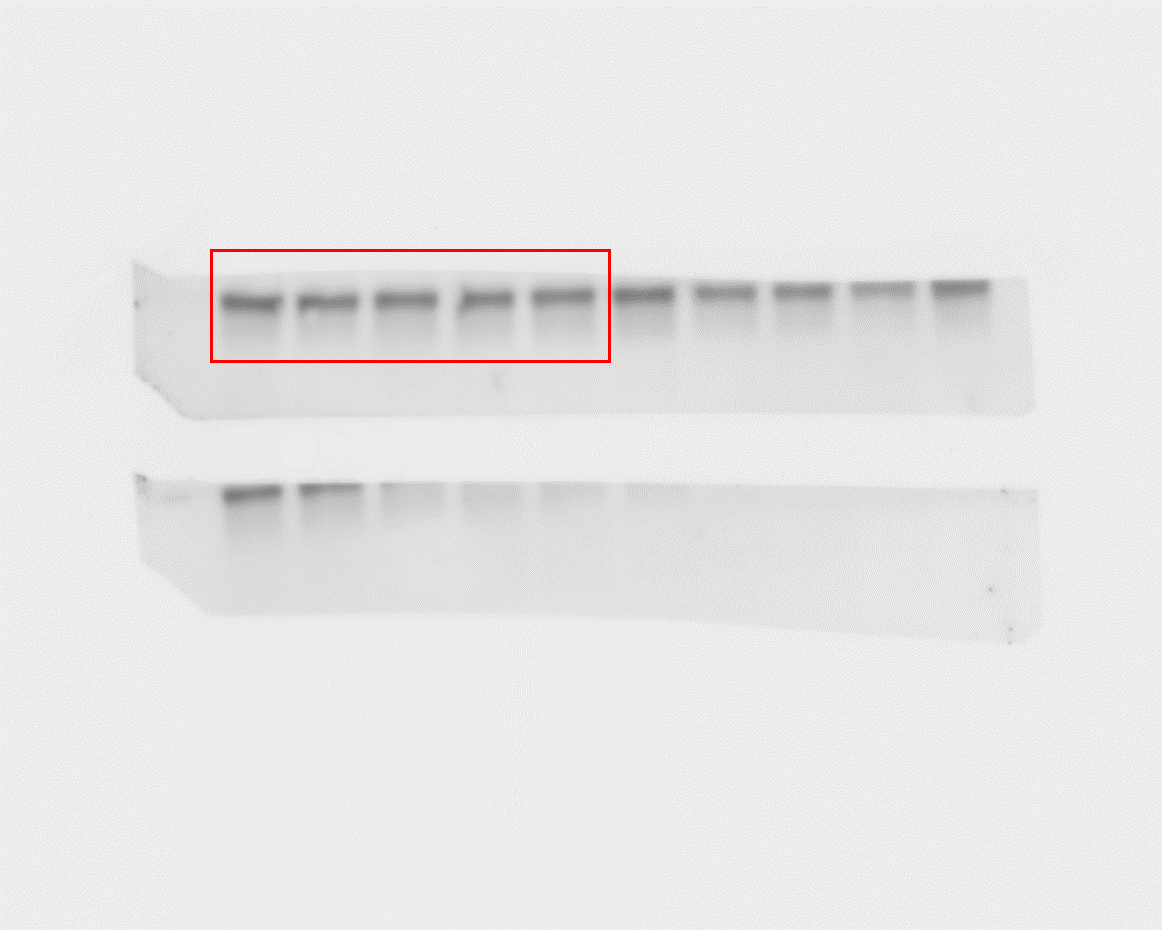


BIM_EL_


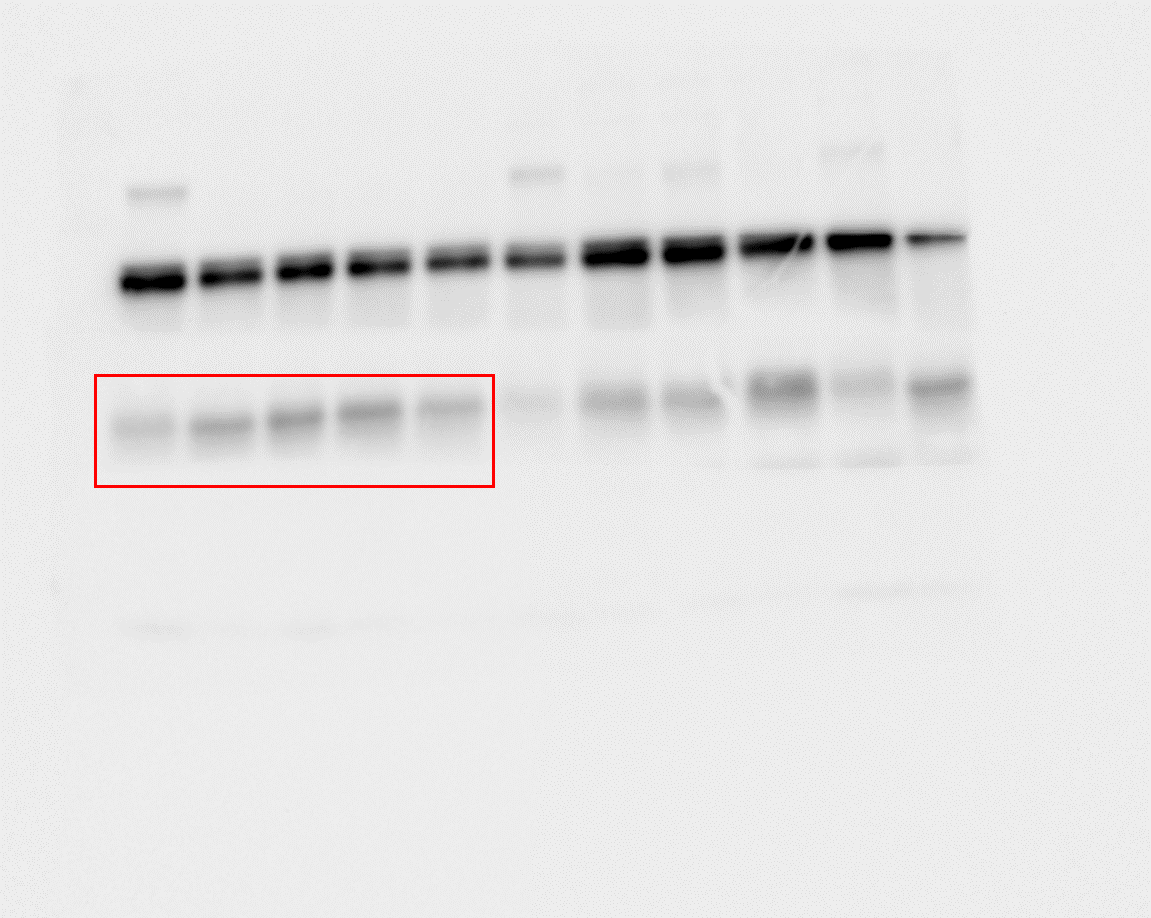


**Figure 3**

H3122 cell line

Tubulin


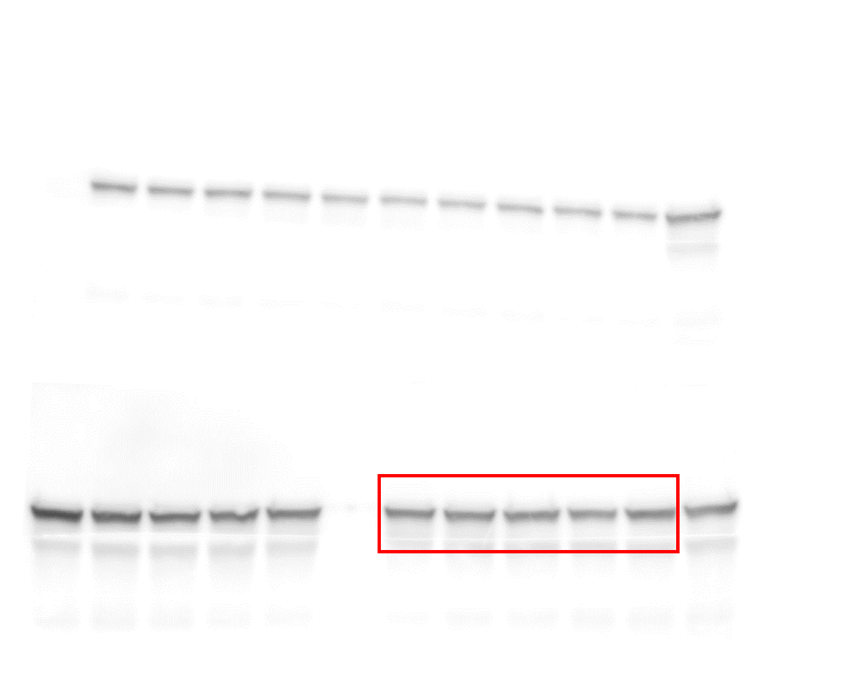


MCL-1


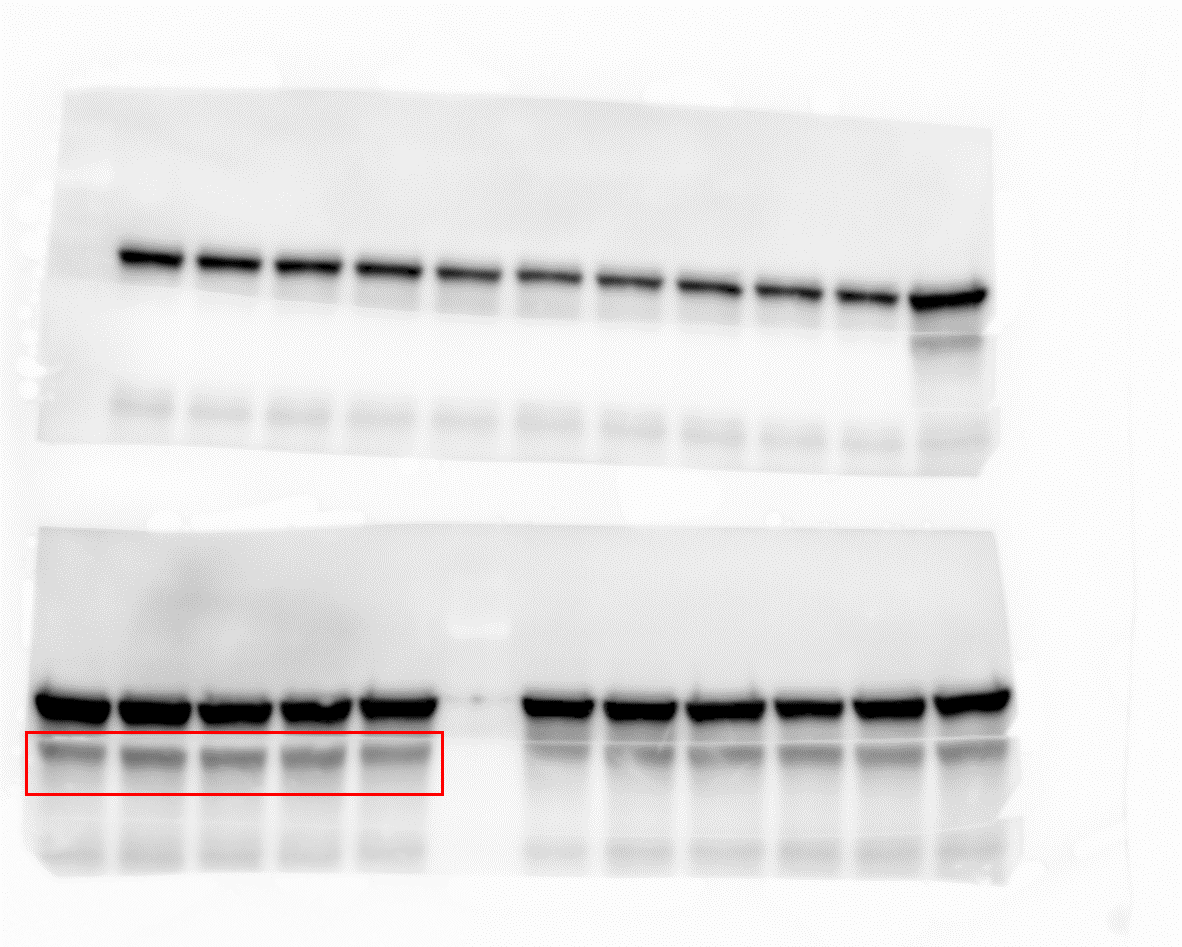


BCL-xL


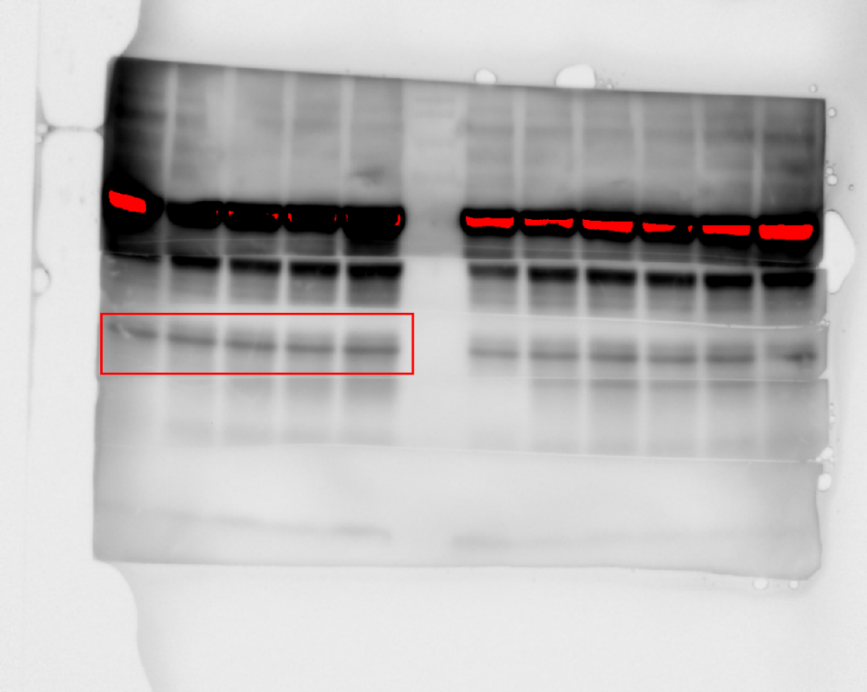


HRK


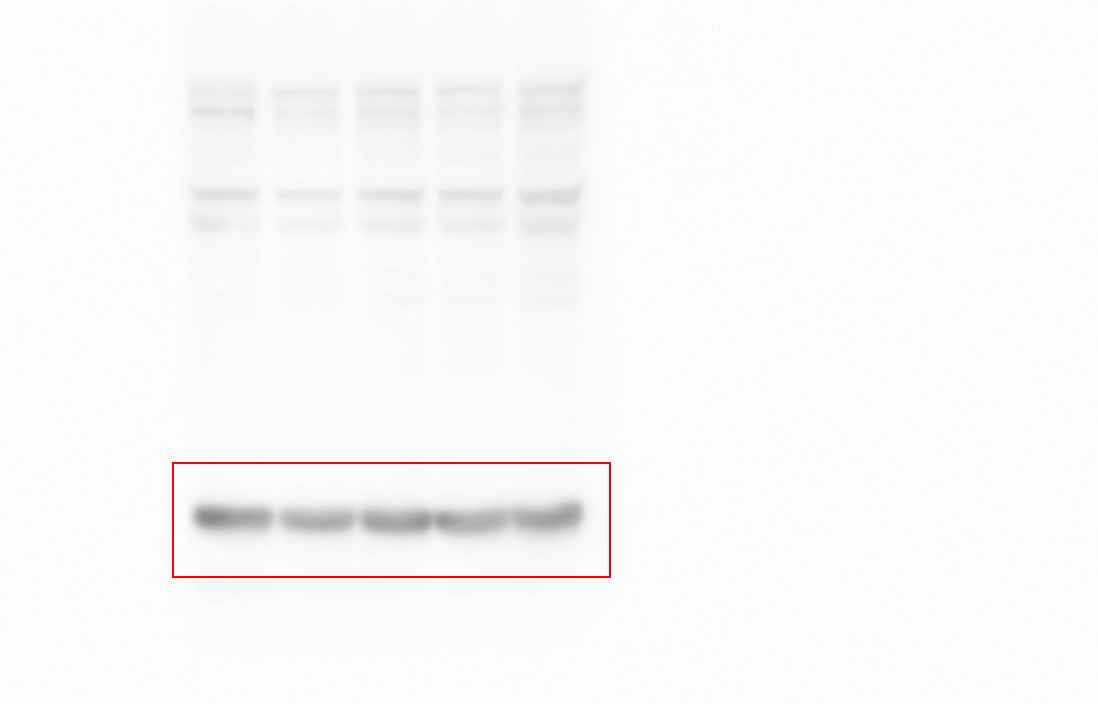


NOXA


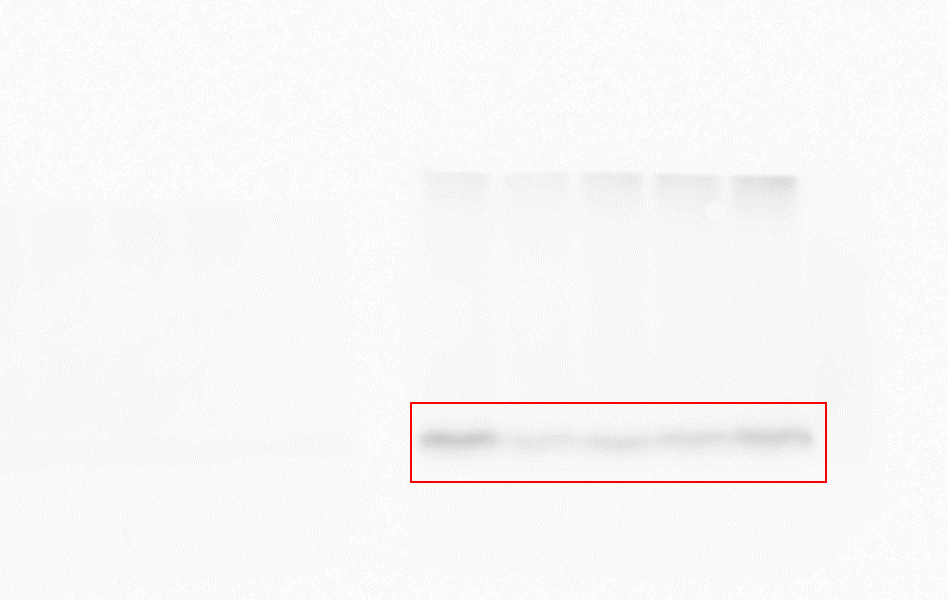


**Figure 4**

H2228 cell line

Tubulin


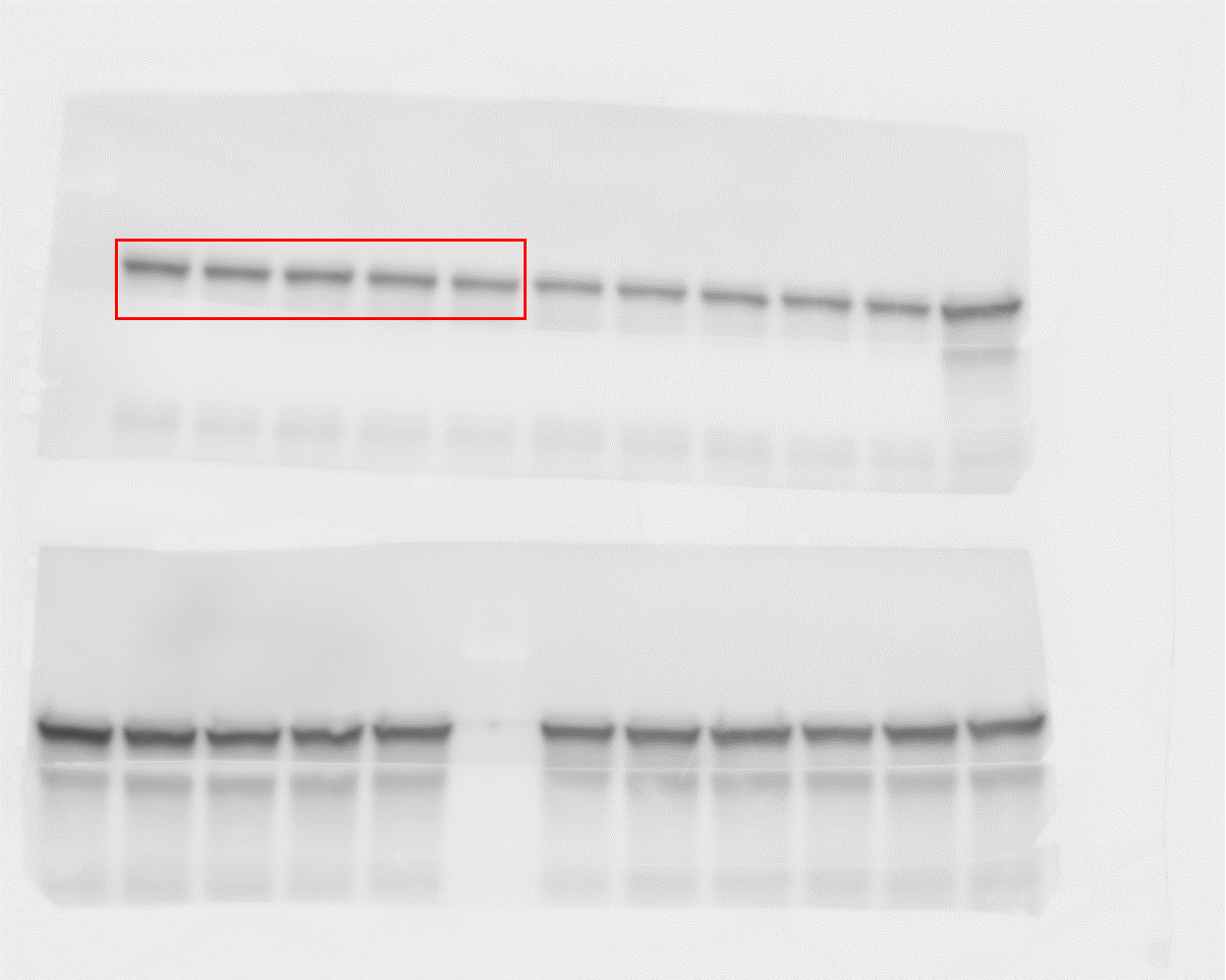


MCL-1


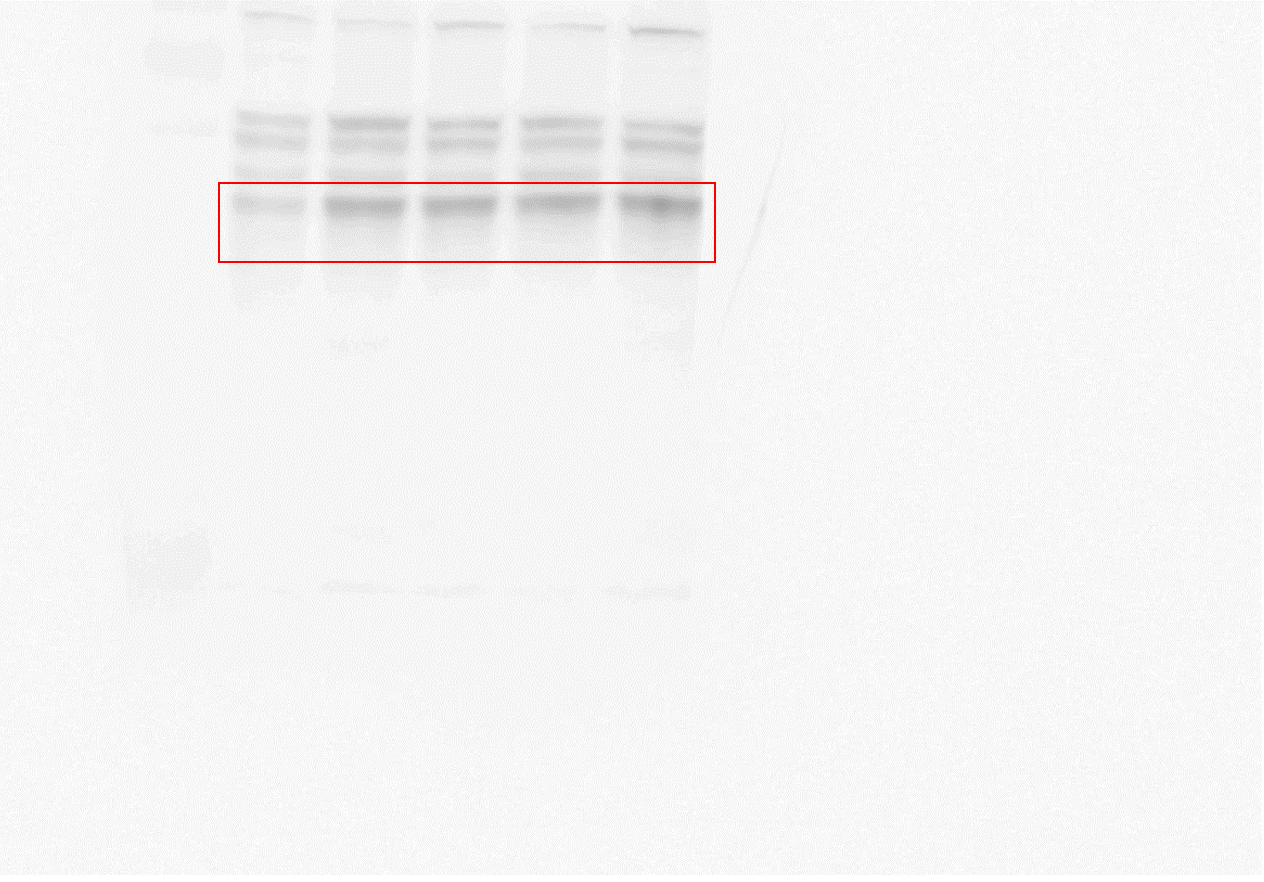


BCL-xL


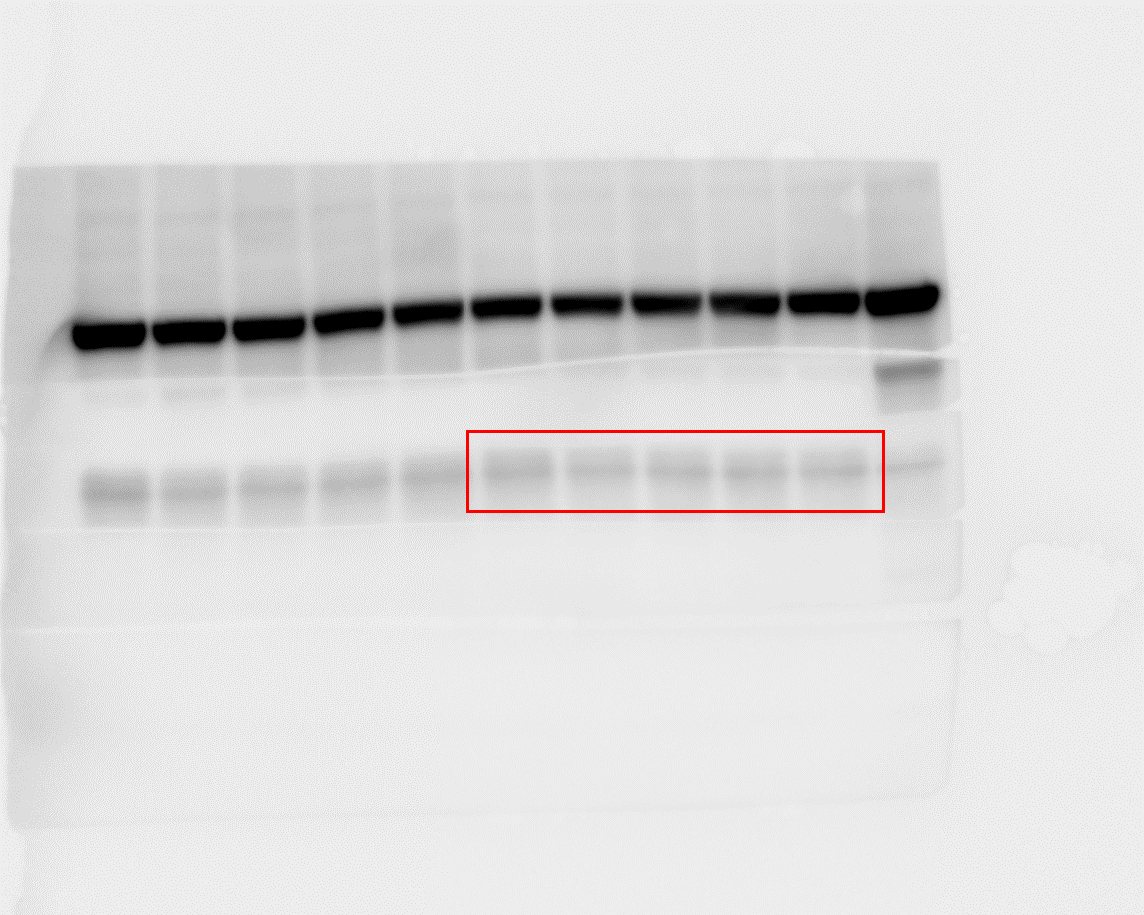


HRK


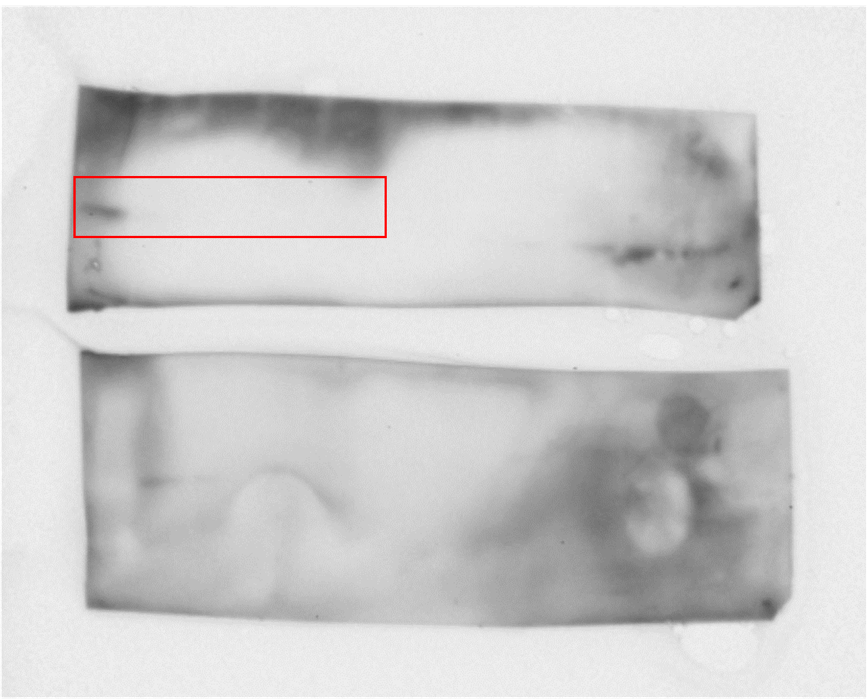


NOXA


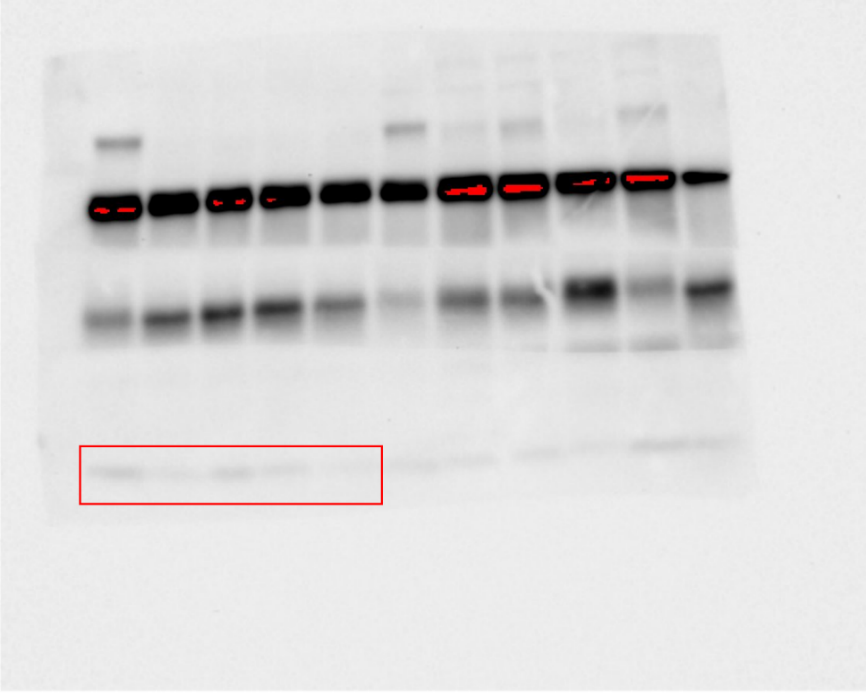


**Figure 5**

H2228 cell line

Tubulin


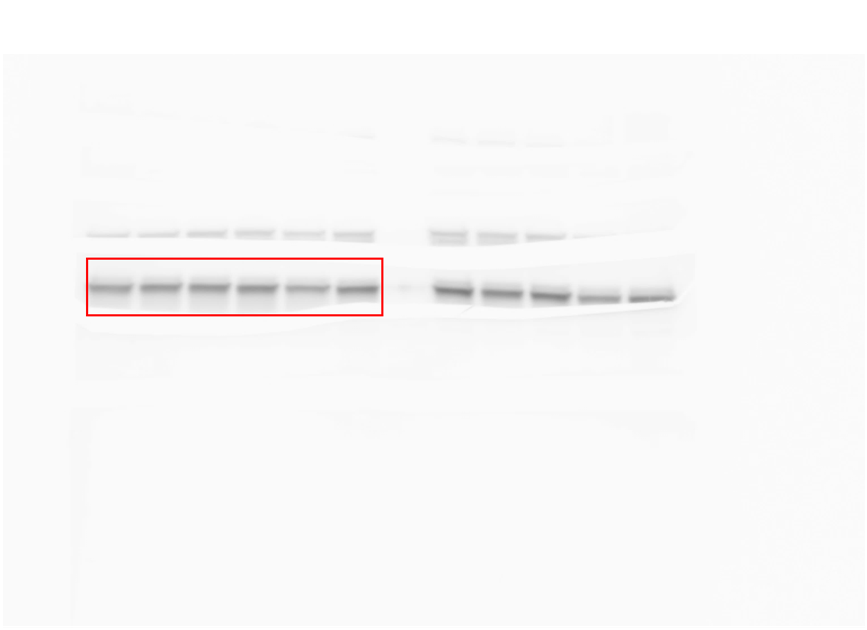


p-AKT


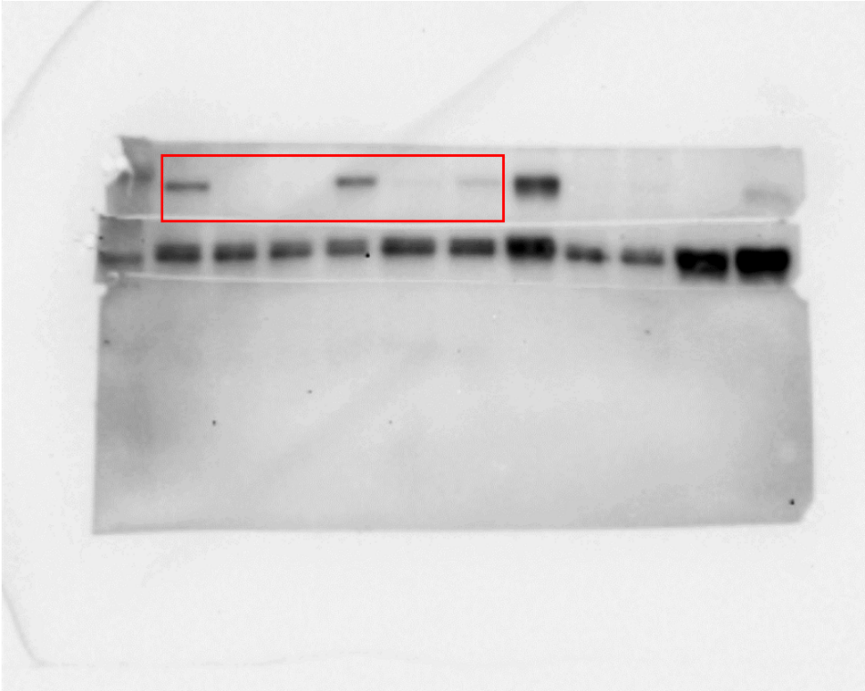


AKT


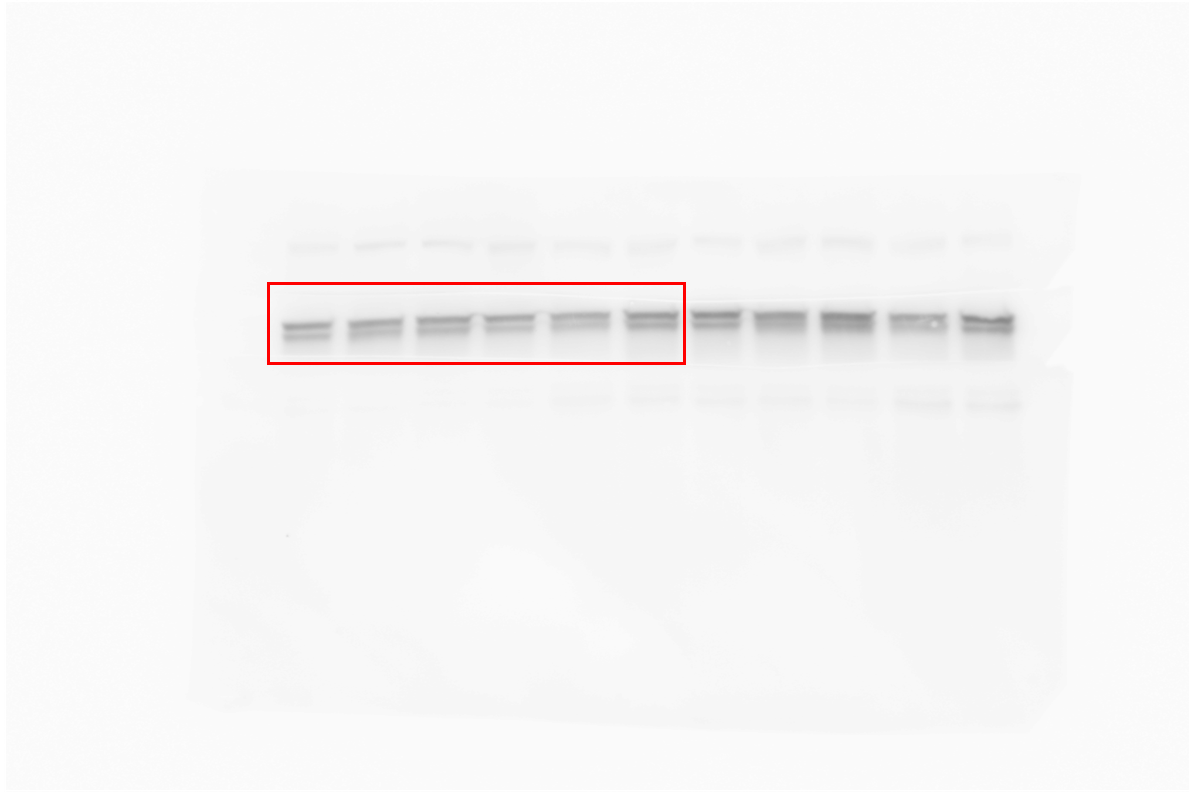


p-ERK


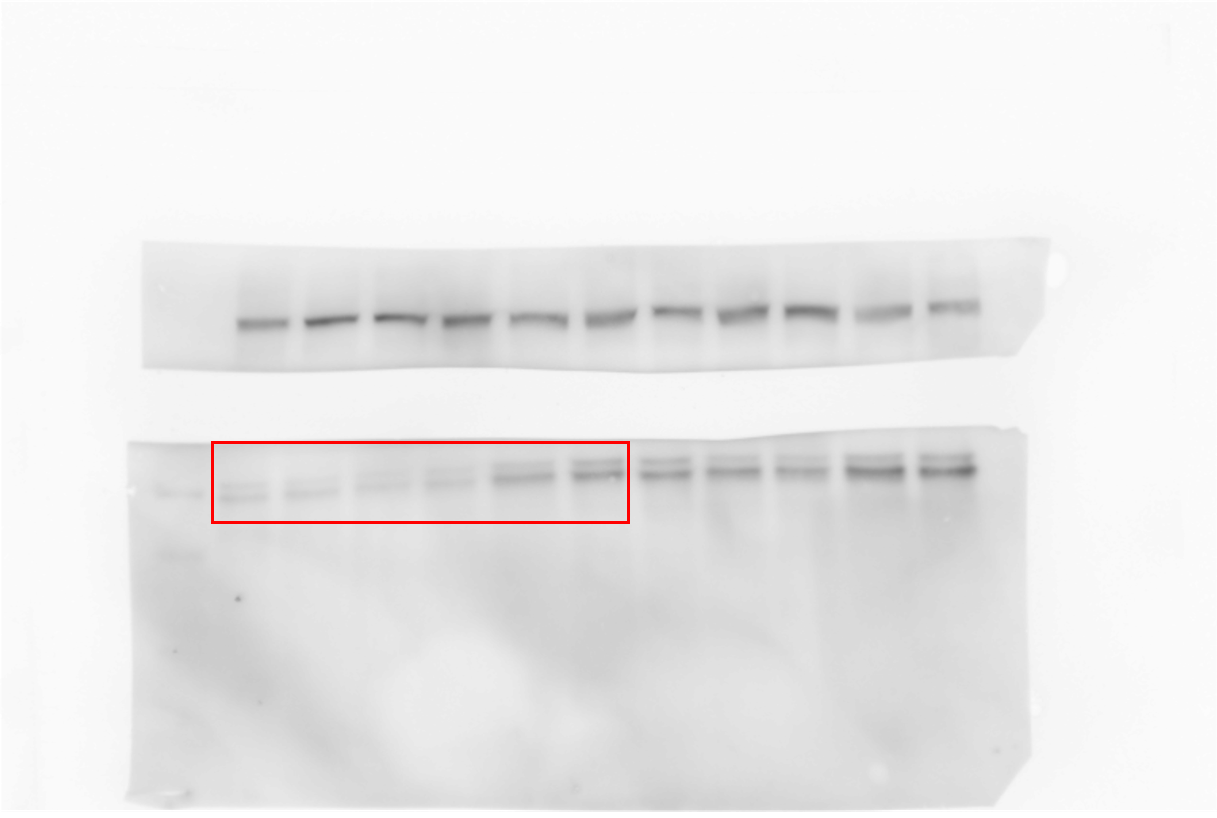


ERK


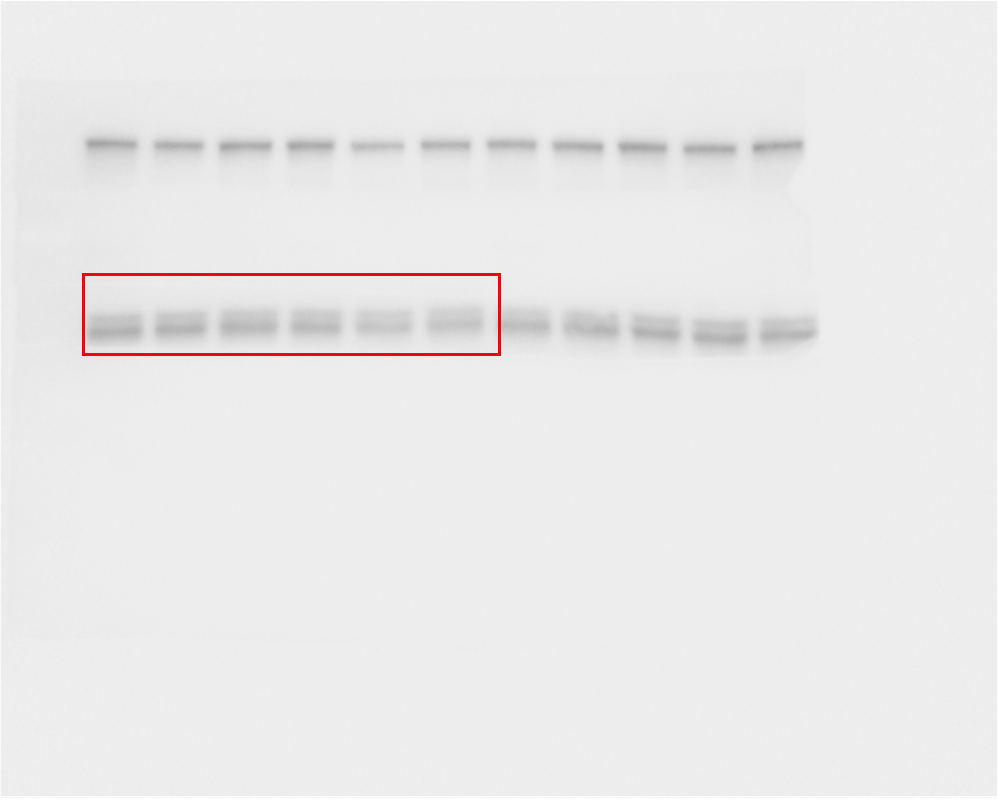


BIM_EL_


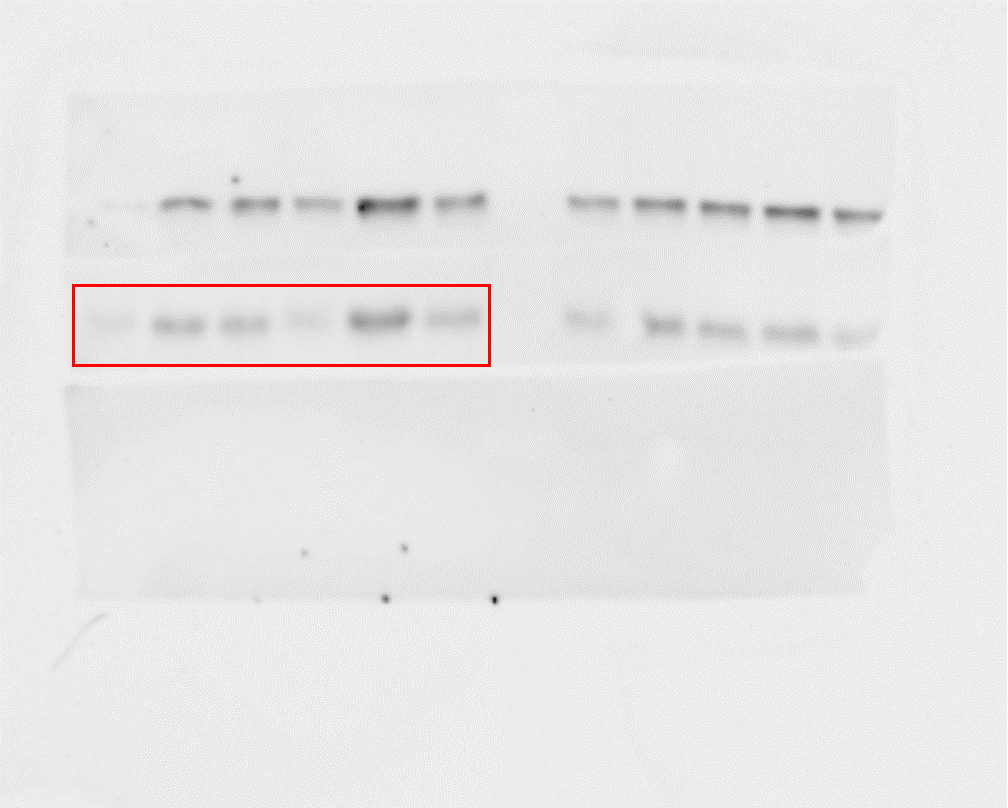

Supplement: Supplementary file 2 — Supplemental Material [file 41419_2025_7513_MOESM2_ESM.docx]
